# Relationships between Root Pathogen Resistance, Abundance and Expression of *Pseudomonas* Antimicrobial Genes, and Soil Properties in Representative Swiss Agricultural Soils

**DOI:** 10.3389/fpls.2017.00427

**Published:** 2017-03-29

**Authors:** Nicola Imperiali, Francesca Dennert, Jana Schneider, Erine Bougouin, Christelle Velatta, Marie Fesselet, Michele Wyler, Fabio Mascher, Olga Mavrodi, Dmitri Mavrodi, Monika Maurhofer, Christoph Keel

**Affiliations:** ^1^Department of Fundamental Microbiology, University of Lausanne Lausanne, Switzerland; ^2^Plant Pathology, Institute of Integrative Biology, Swiss Federal Institute of Technology (ETH) Zurich Zurich, Switzerland; ^3^Plant Breeding and Genetic Resources, Institute for Plant Production Sciences, Agroscope Nyon, Switzerland; ^4^Department of Biological Sciences, University of Southern Mississippi, Hattiesburg MS, USA

**Keywords:** *Pseudomonas*, PGPR, plant-beneficial activity, antimicrobial metabolites, *Pythium ultimum*, *Gaeumannomyces tritici*, soil, disease suppressiveness

## Abstract

Strains of *Pseudomonas* that produce antimicrobial metabolites and control soilborne plant diseases have often been isolated from soils defined as disease-suppressive, i.e., soils, in which specific plant pathogens are present, but plants show no or reduced disease symptoms. Moreover, it is assumed that pseudomonads producing antimicrobial compounds such as 2,4-diacetylphloroglucinol (DAPG) or phenazines (PHZ) contribute to the specific disease resistance of suppressive soils. However, pseudomonads producing antimicrobial metabolites are also present in soils that are conducive to disease. Currently, it is still unknown whether and to which extent the abundance of antimicrobials-producing pseudomonads is related to the general disease resistance of common agricultural soils. Moreover, virtually nothing is known about the conditions under which pseudomonads express antimicrobial genes in agricultural field soils. We present here results of the first side-by-side comparison of 10 representative Swiss agricultural soils with a cereal-oriented cropping history for (i) the resistance against two soilborne pathogens, (ii) the abundance of *Pseudomonas* bacteria harboring genes involved in the biosynthesis of the antimicrobials DAPG, PHZ, and pyrrolnitrin on roots of wheat, and (iii) the ability to support the expression of these genes on the roots. Our study revealed that the level of soil disease resistance strongly depends on the type of pathogen, e.g., soils that are highly resistant to *Gaeumannomyces tritici* often are highly susceptible to *Pythium ultimum* and vice versa. There was no significant correlation between the disease resistance of the soils, the abundance of *Pseudomonas* bacteria carrying DAPG, PHZ, and pyrrolnitrin biosynthetic genes, and the ability of the soils to support the expression of the antimicrobial genes. Correlation analyses indicated that certain soil factors such as silt, clay, and some macro- and micronutrients influence both the abundance and the expression of the antimicrobial genes. Taken together, the results of this study suggests that pseudomonads producing DAPG, PHZ, or pyrrolnitrin are present and abundant in Swiss agricultural soils and that the soils support the expression of the respective biosynthetic genes in these bacteria to various degrees. The precise role that these pseudomonads play in the general disease resistance of the investigated agricultural soils remains elusive.

## Introduction

The ability of soilborne plant pathogens to attack and damage host plants is influenced by biotic and abiotic soil factors (Weller et al., 2002; Haas and Défago, 2005; Lemanceau et al., 2006; Almario et al., 2014). In some soils, even susceptible crop plants suffer only a little or not at all from specific diseases although soilborne pathogens are present (Weller et al., 2002). In general, two different types of natural pathogen suppression are thought to occur in agricultural soils. First, the general disease suppression, where different soilborne pathogens are controlled to a certain degree depending on the total microbial activity in the soil and/or on abiotic soil factors (Weller et al., 2002; Lemanceau et al., 2006). Second, the specific disease suppression, where the soil restricts the activity of a distinct species of plant pathogen based on its interactions with a specific group of microorganisms (Weller et al., 2002; Haas and Défago, 2005; Lemanceau et al., 2006; Berendsen et al., 2012; Raaijmakers and Mazzola, 2016).

Soils with specific disease suppression have been described worldwide (Cook and Rovira, 1976; Stutz et al., 1986; Weller et al., 2002; Lemanceau et al., 2006; Chng et al., 2015) and for diverse soilborne plant pathogens. They include soils suppressive to *Gaeumannomyces graminis var. tritici* (recently renamed *G. tritici* (Hernández-Restrepo et al., 2016)) causing take-all of wheat (Weller et al., 2002), *Thielaviopsis basicola* causing black root rot of tobacco (Stutz et al., 1986; Almario et al., 2014), *Fusarium oxysporum* causing wilt on tomatoes (Alabouvette, 1986; Tamietti et al., 1993), *Pythium* spp. causing seedling damping-off (Martin and Hancock, 1986), and *Rhizoctonia solani* causing damping-off and root rot on various crop species (Mendes et al., 2011). Such soils are commonly referred-to as *suppressive soils*. By contrast, *conducive soils* do not restrict the development of soilborne diseases (Haas and Défago, 2005).

Suppressive soils have been found to host distinct microbial communities that are thought to be responsible for the natural disease control effect (Weller et al., 2002; Haas and Défago, 2005; Mendes et al., 2011, 2013; Klein et al., 2013; Kyselkova et al., 2014; Cha et al., 2016). In particular, bacteria of the *Pseudomonas fluorescens* group have been isolated from suppressive soils and used as plant or soil inoculants. Several strains proved to be very efficient at colonizing roots, protecting plants from different diseases, and increasing plant productivity (Mercado-Blanco and Bakker, 2007; Lugtenberg and Kamilova, 2009; Höfte and Altier, 2010). Thus, it has been suggested that such pseudomonads contribute to soil suppressiveness (Weller et al., 2002; Haas and Défago, 2005; Garbeva et al., 2006; Lemanceau et al., 2006; Weller, 2007; Mazurier et al., 2009). The capacity of many root-associated pseudomonads to release antimicrobial compounds placed them in the focus of research on the nature of soil disease suppressiveness. Many *P. fluorescens* group strains produce an array of potent antimicrobials, among which 2,4-diacetylphloroglucinol (DAPG), phenazines (PHZ), pyrrolnitrin (PRN), and hydrogen cyanide (HCN) are most prominent (Blumer and Haas, 2000; Haas and Défago, 2005; Raaijmakers and Mazzola, 2012; Mavrodi et al., 2013). All these antimicrobials were shown, mostly in pot and gnotobiotic assays, to play indeed an important role in the *Pseudomonas*-mediated protection of plants from soilborne pathogenic fungi and oomycetes (Thomashow and Weller, 1988; Voisard et al., 1989; Keel et al., 1992; Maurhofer et al., 1992; Pierson and Thomashow, 1992; Hwang et al., 2002; Chin-A-Woeng et al., 2003; Weller et al., 2007; Mavrodi et al., 2013).

The role of these antimicrobial compounds in disease-suppressive soils is still not fully understood. They are indeed produced in some field soils, as demonstrated for DAPG in the Quincy take-all decline soil (Raaijmakers et al., 1999) and for PHZ in wheat fields of the Columbia Plateau, USA (Mavrodi D.V. et al., 2012). Several studies performed during the last 15 years aimed at investigating whether disease-suppressive soils are specifically enriched for *Pseudomonas* genotypes producing antimicrobials compared to conducive soils. In fact, in the Pacific Northwest of the USA, DAPG-producing pseudomonads were found to be more abundant in take-all suppressive soils than in adjacent conducive soils (Raaijmakers et al., 1997). However, DAPG producers were not more abundant in *Fusarium* suppressive soils of Châteaurenard (France) than in adjacent conducive soils, in contrast to PHZ-producing pseudomonads, which were more abundant in the suppressive soils (Mazurier et al., 2009). In some studies, the total abundance of DAPG-producing pseudomonads was found to be similar in suppressive and conducive soils (Ramette et al., 2006; Almario et al., 2013a), but suppressive soils harbored distinct genotypes of DAPG producers (Frapolli et al., 2010). Moreover, abundances of plant-beneficial pseudomonads with antimicrobial activity were mostly investigated in specific disease-suppressive soils, and very little is known about the occurrence of these bacteria in common agricultural soils and on how soil factors might impact these bacteria. In wheat fields of the Pacific Northwest of the USA, it was found that the abundance of DAPG- and PHZ-producing pseudomonads on wheat roots is influenced by irrigation (Mavrodi O.V. et al., 2012).

Based on such observations, the abundance and genotypic diversity of antimicrobials-producing *Pseudomonas* bacteria in soil seems not a sufficient argument to explain the disease suppressiveness of some soils. It has been suggested that (i) other bacterial species contribute importantly to the disease suppressiveness (Mendes et al., 2011; Kyselkova et al., 2014), and (ii) somehow the expression of antimicrobial genes in *Pseudomonas* bacteria is favored in suppressive soils and hampered in conducive soils (Ramette et al., 2006; Almario et al., 2014). Indeed, studies on the abundance of antimicrobial metabolite-producing pseudomonads do not consider the complex interactions in the rhizosphere that ultimately modulate the production of the antimicrobials in the rhizosphere (Rochat et al., 2010; de Werra et al., 2011). To date, little is known about the biotic and abiotic factors affecting the expression of biosynthetic genes for these metabolites in soil. Studies conducted under gnotobiotic conditions indicate that the expression of DAPG, HCN, and PRN biosynthesis genes is influenced by the crop species and variety (Rochat et al., 2010; de Werra et al., 2011; Latz et al., 2015) and for DAPG also by the interaction with other microorganisms and the iron availability in the rhizosphere (Notz et al., 2001, 2002; Maurhofer et al., 2004; Jousset et al., 2010, 2011; Almario et al., 2013b). How biotic and abiotic soil factors affect antimicrobial gene expression under natural conditions in agricultural soils remains, however, unexplored.

There is a clear lack of studies investigating the link between natural disease resistance and abundance and expression of antimicrobial *Pseudomonas* genes in common agricultural soils. To address this gap, in the present study 10 representative Swiss agricultural soils with a cereal-oriented cropping history and differing in their physical and chemical characteristics were compared for their resistance to two soilborne pathogens of wheat, i.e., *G. tritici* (Gt) and *Pythium ultimum* (Pu). In parallel, the 10 soils were planted with wheat and pseudomonads harboring the biosynthetic genes required for the production of the antimicrobial compounds DAPG, PHZ, and PRN were quantified on roots using qPCR. In addition, the expression of these genes and the HCN biosynthetic genes was monitored by flow cytometry using fluorescent reporter strains of the representative model pseudomonads *P. protegens* CHA0 and *P. chlororaphis* PCL1391. To our best knowledge, this is the first side-by-side comparison using root-associated pseudomonads as bio-indicators to explore relationships between abundance and expression of antimicrobial genes, soil disease resistance and soil physicochemical characteristics in a range of common agricultural soils.

## Materials and Methods

### Sampling and Physicochemical Analysis of Field Soils

**FIGURE 1:**
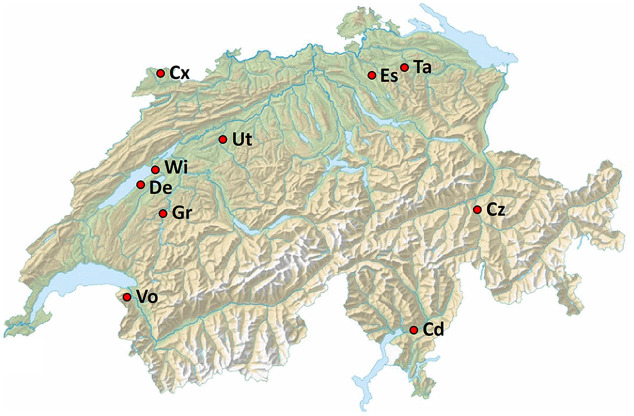
**Location of the 10 field sites in Switzerland used for soil sampling**. Cd, Cadenazzo; Cx, Courtedoux; Cz, Cazis; De, Delley; Es, Eschikon; Gr, Grangeneuve; Ta, Taenikon; Ut, Utzenstorf; Vo, Vouvry; Wi, Witzwil. Empty topographical map of Switzerland courtesy of Federal Office of Topography swisstopo.

Soil samples were collected in 10 farmer’s fields across Switzerland (**Figure 1**) in May 2013. The main characteristics of the 10 field soils are listed in **Table 1**. Field sites had a history of multi-year cereal-oriented crop rotation and were chosen to represent predominant Swiss agricultural soil types and climate conditions. All fields were cropped with winter wheat in the year of sampling. For sampling, soil cores of 15–20 cm depth were extracted with disinfected soil recovery augers between the rows of wheat plants at 20 random locations in each field in order to obtain a representative sample. Then, extracted soil samples were sieved (mesh size, 10 by 10 mm) in order to remove stones, plant residues or other larger material, pooled and thoroughly mixed. For each site, approximately 120 kg of sieved soil were collected and stored in barrels for 3 months at 15°C before the start of the experiments in order to equilibrate the soils, i.e., to minimize effects of different environmental conditions (e.g., temperature, soil moisture) prevailing at the different sampling sites at the time of sampling. The storage temperature was chosen because it can be considered as the average temperature in Switzerland during the growing season of wheat from April to September (according to long-term monthly temperature averages recorded by the Swiss Federal Office MeteoSwiss^1^). Soil parameter analyses were carried out by the Labor für Boden- und Umweltanalytik (Eric Schweizer AG, Steffisburg, Switzerland) following standard protocols used in Swiss agriculture (Agroscope, 2006). Concentrations of soluble, readily plant-available macronutrients were determined following H_2_O extraction, for which soil samples were suspended in distilled water at a ratio of 1:10 (g mL^-1^). Reserve macronutrients and micronutrients were extracted with ammonium acetate EDTA, for which soil samples dried at 65°C were suspended at a ratio of 1:10 (g mL^-1^) in a solution consisting of acetic acid (0.5 mol L^-1^), ammonium acetate and EDTA (0.02 mol L^-1^) adjusted to a pH of 4.65. Soil suspensions were then vigorously shaken for 1 h and filtered prior to analysis by mass spectrometry. The soil parameter analyses were carried out (i) directly after sampling and (ii) after the end of the experiments. Except for nitrate, the soil parameters did not change significantly between the two analyses (**Table 1**).

**Table 1:** Characteristics of agricultural soils sampled at different Swiss farmers’ fields cropped with wheat^1^.

	Cadenazzo (Cd)	Courtedoux (Cx)	Cazis (Cz)	Delley (De)	Eschikon (Es)	Grangeneuve (Gr)	Tänikon (Ta)	Utzenstorf (Ut)	Vouvry (Vo)	Witzwil (Wi)
Coordinates^2^										
SN	46.160862	47.405785	46.75664	46.918324	47.447086	46.774855	47.482525	47.11437	46.330365	46.983912
EW	8.934138	7.027313	89.423094	6.964087	8.687439	7.112180	8.910636	7.565597	6.904972	7.071365
Basic soil properties										
Organic matter (%)	1.7	2.6	2.2	1.7	2.4	1.7	3.5	4.7	1.5	11.6
Clay (%)	8.4	25.7	7.8	16.8	24.1	19.2	27.0	20.4	7.4	31.0
Silt (%)	42.9	61.4	32.9	26.7	27.9	16.0	24.0	32.7	33.1	30.5
Sand (%)	47.8	10.5	58.4	55.7	46.7	64.3	47.1	44.3	59.0	30.5
pH	6.0	6.7	7.7	7.3	7.0	6.6	6.9	7.0	7.1	7.3
Soluble macronutrients (H_2_O, 1:10)^3^										
Nitrate (analysis 1)^4^ (mg kg^-1^)	8.2	12.6	26.7	23.1	34.6	29.4	34.3	30.3	10.0	52.0
Nitrate (analysis 2)^4^ (mg kg^-1^)	80.9	10.1	153.9	21.8	192.8	9.1	148.0	80.1	77.7	91.9
P (mg kg^-1^)	4.3	2.2	1.9	6.0	1.5	2.5	3.6	7.0	4.2	1.1
K (mg kg^-1^)	3.7	18.3	22.2	30.7	22.3	14.8	43.4	56.8	14.9	35.3
Ca (mg kg^-1^)	36.4	164.8	155.7	144.6	117.4	76.9	118.1	135.6	103.5	332.2
Mg (mg kg^-1^)	5.2	8.2	14.7	10.8	14.0	11.5	21.8	15.9	7.3	14.2
Reserve macronutrients (NH_4_-Ac. + EDTA, 1:10)^3^									
P (mg kg^-1^)	41.5	45.9	77.9	211.5	29.5	38.4	206.6	291.7	137.3	33.7
K (mg kg^-1^)	46.6	182.2	50.5	140.8	110.9	98.9	261.1	240.9	50.5	81.6
Ca (mg kg^-1^)	870.1	4608.0	21880.0	3386.0	2611.0	1746.0	4122.0	3927.0	27170.0	69060.0
Mg (mg kg^-1^)	79.7	98.5	232.6	93.9	141.3	116.6	392.5	191.8	256.1	537.3
Micronutrients (NH_4_-Ac. + EDTA, 1:10)^3^									
Fe (mg kg^-1^)	248.2	336.5	552.1	836	381.4	372.8	1385.0	1275.0	785.0	1500.0
Cu (mg kg^-1^)	8.0	5.7	34.8	11.3	8.4	6.6	15.4	16.2	23.6	17.7
B (mg kg^-1^)	0.0	0.3	0.4	0.8	0.1	0.0	0.6	0.8	1.0	0.9
Mn (mg kg^-1^)	148.8	790.8	754.9	528.3	686.7	601.2	911.1	682.4	184.9	207.5
Zn (mg kg^-1^)	1.9	6.1	9.25	4.51	7.0	3.3	11.6	14.3	4.3	14.7

^1^*Soils from farmers’ fields were sampled in May 2013. All fields were planted with winter wheat at the time of sampling. Soil parameters were analyzed by the Labor für Boden- und Umweltanalytik, Eric Schweizer AG, Steffisburg, Switzerland after sampling in 2013*.

^2^Geographical coordinates of each field site were measured by GPS. The field site localization was described by the south–north orientation (SN) and the east–west (EW) orientation codes.

^3^Souble macronutrients were extracted with water. Reserve macronutrients and micronutrients were extracted with ammonium acetate EDTA. See section “Materials and Methods” for details.

^4^Soil parameters were analyzed twice, once immediately after sampling and once at the end of the experiments, i.e., after prolonged storage. Only nitrate levels (shown in the table) were significantly different between the first and the second analysis.

### Assessment of Field Soil Resistance against Root Pathogens

In order to test the natural resistance of the 10 Swiss agricultural soils toward soilborne pathogens, pot experiments were carried out in the greenhouse with cucumber and Pu and wheat and Gt. The Pu inoculum was prepared by inoculating 25 g of autoclaved millet seeds moistened with 10 mL of sterile water with three plugs of a culture of Pu strain ETH-2 (isolated from a Swiss agricultural soil) grown on Oxoid malt agar (Thermo Fisher Scientific, Reinach, Switzerland) for 7 days. Pu millet seed cultures were grown for 7 days at 18°C and then cut into small fragments for soil inoculation. The Gt inoculum was prepared by inoculating 100 g of autoclaved oat seeds without spelts and moistened with 100 mL of sterile, distilled water with 20 plugs of cultures of Gt strain I-17 (Lebreton et al., 2007) grown on Oxoid potato dextrose agar for 14 days. Gt oat cultures were grown for 4 weeks at 24°C in the dark and then dried in a sterile cabinet on sterile filter paper for 3 days. Seeds of cucumber (*Cucumis sativus* cv. Chinese Snake) and spring wheat (*Triticum aestivum* cv. Rubli) were surface-sterilized for 30 min in 1.5% (v/v) NaOCl, rinsed with sterile distilled water and pre-germinated on sterile moist filter paper for 2 days in the dark at 24°C. For the plant experiments, 250-mL plastic pots of were filled with a 4:1 mixture (wt/wt) of the respective field soil and quartz sand (grain size of 0.5–2.2 mm diameter). Pathogen inoculum at different concentrations was thoroughly mixed into soil. Pu inoculum was added at 0.125, 0.25, 0.5, or 1.0 g inoculum per pot, whereas Gt inoculum was added at 0.2, 0.6, 2.0, or 6.0 g per pot. Pots in control treatments contained field soil without pathogen addition. Three seedlings of cucumber or wheat, respectively, were then planted per pot. For each treatment, six replicate pots were prepared. Plants were grown in the greenhouse at 70% relative humidity with light (210 μmol m^-2^ sec^-1^) for 16 h at 22°C (cucumber) or 18°C (wheat), followed by an 8-h dark period at 18°C (cucumber) or 15°C (wheat). Plants were watered routinely to keep the soil at constant moisture. The position of the pots was changed at random every other day to avoid position effects. After incubation for 10 days (cucumber) or 21 days (wheat), total shoot fresh weights per pot were assessed.

### Development of qPCR Methods for Quantification of Pseudomonads Harboring DAPG, PHZ, and PRN Biosynthetic Genes

To quantify the abundance of DAPG and PHZ producing bacteria in soil, we developed quantitative real-time polymerase chain reaction (qPCR) assays targeting *phlD* and *phzF* genes. These genes encode, respectively, a polyketide synthase involved in the synthesis of phloroglucinols from malonyl-CoA (Bangera and Thomashow, 1996; Achkar et al., 2005) and an isomerase involved in the synthesis of phenazine-1-carboxylic acid (Mavrodi et al., 1998; Blankenfeldt et al., 2004). Alignments were created with publicly available *phlD* and *phzF* sequences from GenBank^2^ and conserved regions were chosen for the design of primers and probes (**Table 2**), which was carried out with the Primer 3 Plus software (Untergasser et al., 2007). The parameters were amplicon length between 100 and 200 bp, melting temperature (TM) between 50 and 70°C, TM of probe 5°C higher than TM of primers, and the default setting of the program for self-complementarity and 3′-end stability. Partial sequences of *phlD* (GenBank accession CP003190.1| :6563260-6563937) of strain *P. protegens* CHA0 (Jousset et al., 2014) and *phzF* (locus tag, PFLU3_RS28075) of *P. synxantha* 2–79 (Nesemann et al., 2015) were used for primer design. The specificity of the primers was tested *in silico* with Primer-Blast (Ye et al., 2012) and *in vitro* with genomic DNA from 28 DAPG-producing strains and 38 PHZ-producing strains of the *P. fluorescens* group and nine additional PHZ-producing strains (Supplementary Table S1). Results of these tests revealed that our qPCR assays amplify *phlD* and *phzF* genes exclusively from DAPG and PHZ producing species of the *P. fluorescens* lineage. The PRN biosynthetic genes were quantified on wheat roots by the qPCR method of Garbeva et al. (2004). That assay targets a gene for the class IA oxygenase PrnD that is involved in the final step of PRN biosynthesis (Kirner et al., 1998). In contrast to our *phlD* and *phzF* primers, the primers of Garbeva et al. (2004) have broader specificity and, in addition to *Pseudomonas*, amplify *prnD* from PRN-producing strains of *Burkholderia* and *Serratia*.

**Table 2:** Primers and probes used to quantify antimicrobial genes with qPCR.

Metabolite, target gene	Primers and probes^1^	Sequence (5′–3′)	Annealing temperature (°C)	Reference
DAPG^2^, *phlD*	PhlD_65F_DEG	GGT RTG GAA GAT GAA RAA RTC	50	This study; Flury et al., 2017
	PhlD_153P_DEG	FAM-ATG GAG TTC ATS ACV GCY TTG TC-BHQ1		
	PhlD_236R_DEG	GCC YRA BAG YGA GCA YTA C		
Phenazine*, phzF*	PhzF_2Fm	ACC GGC TGT ATC TGG AAA CC	62	This study
	PhzF_2Pm	FAM-GCC GCC AGC ATG GAC CAG CCG AT-BHQ1		
	PhzF_2Rm	TGA TAG ATC TCG ATG GGA AAG GTC		
Pyrrolnitrin, *prnD*	PrnD_F	TGC ACT TCG CGT TCG AGA C	60	Garbeva et al., 2004
	PrnD_P	FAM-CGA CGG CCG TCT TGC GGA TC-BHQ1		
	PrnD_R	GTT GCG CGT CGT AGA AGT TCT		
Internal control, APA9 plasmid^3^	CMV_1F	TCA TCA TTT CCA CTC CAG GCT C	62	Von Felten et al., 2010
	CMV_1R	TCA TCC CTC TGC TCA TAC GAC TG		

^1^*TaqMan probes were labeled with fluorescein (FAM) at the 5′ end and with the black hole quencher 1 (BHQ-1) at the 3′ end*.

^2^*DAPG, 2,4-diacetylphloroglucinol*.

^3^*Plasmid from cassava mosaic virus*.

The efficiency of *phlD* and *phzF* primers at low gene copy numbers was evaluated using *in vitro* standard curves prepared by serially diluting genomic DNA of *P. protegens* CHA0 and *P. synxantha* 2–79. The genomic DNA was prepared by growing both strains in lysogeny broth (LB) (Bertani, 1951) overnight at 24°C on a rotary shaker at 180 rpm and extracting DNA with the Wizard Genomic DNA Purification Kit (Promega AG, Dübendorf, Switzerland). The concentration of purified DNA was quantified by fluorimetry with Qbit (Thermo Fisher Scientific). We also generated an *in vivo* standard curve for each qPCR assay to quantify the corresponding target genes on wheat roots. To this end, aliquots of 1 g of 21-days-old roots of spring wheat cv. Rubli grown in autoclaved soil were inoculated with decreasing concentrations of a mixture of bacterial cells belonging to different strains carrying the respective target gene. Strains used for *in vivo* standard curves are listed in Supplementary Table S1. Bacterial cells were harvested from overnight cultures in LB, washed and suspended in sterile 0.9% NaCl solution. Cell suspensions from each strain were set to the same optical density at 600 nm (OD_600_) and then mixed together at equal proportions. The mixed suspensions were adjusted to an OD_600_ of 0.125, corresponding to approximately 10^8^ CFU mL^-1^, serially diluted and inoculated at 10^1^, 10^2^, 10^3^, 10^4^, 10^5^, 10^6^, 10^7^ and 10^8^ CFU g^-1^ roots for the preparation of the standard curve. For each concentration and for the control without bacteria, three replicates were performed. The inoculated root samples used for standard curves were processed with the same method as the samples from pot experiments with the different soils (see following chapter). *In vivo* standard curves were prepared as described above for the *phlD*, *phzF* and *prnD* qPCR, using strains listed in Supplementary Table S1. Since all *in vivo* standard curves were prepared with bacterial cells recovered from wheat roots, the CT values can be directly converted to numbers of bacteria harboring *phlD*, *phzF*, or *prnD* per g root. Our qPCR data also directly reflect the abundance of the antimicrobial biosynthesis genes because *phlD, phzF*, or *prnD* are present in single copy in genomes of the *P. fluorescens* group (Flury et al., 2016). A survey of published bacterial genomes revealed that *phzF* and *prnD* are also found as a single copy in other bacterial species such as *Burkholderia* (*phzF* and *prnD*), *Pectobacterium* (*phzF*) or *Serratia* (*prnD*).

### qPCR-Based Quantification of Antimicrobial Genes on Roots of Wheat Grown in Field Soils

To standardize the root material for qPCR quantification of DAPG, PHZ and PRN biosynthetic genes, soil samples from the 10 Swiss field sites were planted with spring wheat cv. Rubli in a greenhouse pot experiment. Plastic pots of 8 cm diameter and 30 cm height were part-filled with field soil and three wheat seedlings, prepared as described above, were planted per pot. Six pots per field soil were prepared. Wheat plants were grown for 2.5 months under the conditions described above for the Gt resistance assays. Root samples were collected, rinsed with tap water, incubated overnight in a sterile 0.9% NaCl solution at 3°C, and then vigorously agitated at 350 rpm for 30 min. Roots were separated from the root-wash suspensions and kept for dry weight assessment. Root-wash suspensions were centrifuged at 3500 rpm for 20 min. The supernatant was discarded and aliquots of 0.5 ml of the resulting root-wash pellet were used for DNA extraction. To each sample, 10^9^ copies of the APA9 plasmid from a cassava mosaic virus were added as an internal standard (Von Felten et al., 2010). DNA extraction was performed with the MPBio soil kit (MP Biomedicals, Illkirch, France) following the protocol of the manufacturer. The concentration of extracted DNA was measured with Qbit. qPCR reactions consisted of 10 μL TaqMan Gene Expression Master Mix (Applied Biosystems, Foster City, CA, USA), 2 μL of the respective forward and reverse primer solutions (10 μM), 2 μL of the respective probe solution (2.5 μM), 0.5 μL of bovine serum albumin solution (20 mg mL^-1^), and 2 μL of template DNA in a total reaction volume of 20 μL. Primer and probe sequences are indicated in **Table 2**. Cycling conditions consisted of 2 min at 50°C (to permit uracil-DNA glycosylase activity), an initial denaturation step of 10 min at 95°C, and 40 cycles of 15 s at 95°C, 30 s at annealing temperature (see **Table 2**) and 30 s at 72°C. In all samples, the added APA9 plasmid was quantified with the primers listed in **Table 2**, following the method of Von Felten et al. (2010). The results from the APA9 plasmid quantification were used to normalize DNA extraction.

To compare the abundance of *phlD* and *phzF* measured with qPCR with the abundance measured using a cultivation-dependent terminal endpoint dilution assay, also called most probable number PCR (MPN-PCR) method (Ramette et al., 2006), a greenhouse experiment was carried out with soil samples from the Cazis and Taenikon field sites and spring wheat cv. Rubli cultivated under the same conditions as described above. After 3 weeks, *phzF* and *phlD* qPCR assays were performed on one fraction of each harvested root-wash pellet as described above, while the other fractions were serially diluted into microtiter plate wells (200 μl volume) containing King’s medium B (King et al., 1954) broth amended with 100 mg L^-1^ cycloheximide, 13 mg L^-1^ chloramphenicol and 40 mg L^-1^ ampicillin. The microtiter plates were incubated at 24°C for 3 days and MPN-PCR was performed as described by Ramette et al. (2006), except for adapting annealing temperatures to the primers used in the present study (**Table 2**).

### Quantification of Resident Pu and Gt by qPCR

*Pythium ultimum* and Gt populations naturally present on roots of wheat were quantified using the qPCR methods developed by Cullen et al. (2007) and Bithell et al. (2012), respectively. The reaction mix was prepared as described above for antimicrobial genes, and cycling conditions were set as described previously (Cullen et al., 2007; Bithell et al., 2012). *In vitro* standard curves were performed with genomic DNA of Pu isolate ETH-2 (concentration range from 0.1 ng to 200 ag per reaction) and of Gt isolate I-17 (10 ng to 10^-4^ ng per reaction). Genomic DNA of the two pathogens was extracted with the DNeasy plant mini kit (Qiagen, Hombrechtikon, Switzerland) from lyophilized mycelia prepared from cultures grown in potato dextrose broth (Difco, Becton, Dickinson and Company, Franklin Lakes, USA) for 7 days at 24°C with agitation at 180 rpm.

### Construction and Culture of *Pseudomonas* Reporter Strains

Bacterial strains and plasmids used for generation of *Pseudomonas* reporter strains for monitoring antimicrobial gene expression are listed in **Table 3**. *Pseudomonas* and *Escherichia coli* strains were routinely cultured at 30 and 37°C, respectively, on nutrient agar plates, in LB and in nutrient yeast broth (Stanisich and Holloway, 1972). When appropriate, selective antibiotics were added to the media at the following concentrations: ampicillin, 100 μg mL^-1^; chloramphenicol, 50 μg mL^-1^; gentamicin, 10 μg mL^-1^; kanamycin, 25 μg mL^-1^; and tetracycline, 125 μg mL^-1^. Genomic DNA from *P. protegens* strain CHA0 and *P. chlororaphis* strain PCL1391 was isolated as previously described (Schnider-Keel et al., 2000). Plasmids were extracted and purified using the QIAprep Spin Miniprep kit (Qiagen) or the JETStar Plasmid Purification Midi kit (Genomed, Basel, Switzerland). PCRs were done using the PrimeSTAR HS DNA polymerase kit (Takara Bio Inc., Shiga, Japan) as described elsewhere (Péchy-Tarr et al., 2005). All DNA digestion and ligation reactions were done using standard techniques (Sambrook and Russell, 2001; Schnider-Keel et al., 2001). DNA extractions from agarose gels were carried out with the QIAquick Gel Extraction kit (Qiagen). Transformations of electro-competent cells with plasmid or purified ligation products were performed by electroporation (Schnider-Keel et al., 2000). To amplify genomic DNA or to detect the presence of recombinant DNA in *E. coli* colonies by screening, 100–200 ng of DNA were amplified using the GoTaq DNA polymerase kit (Promega, Dübendorf, Switzerland). All PCR constructs intended for transformation were verified by sequence analysis. DNA sequencing was carried out by GATC Biotech AG (Konstanz, Germany). Sequences were analyzed using the DNASTAR Lasergene software package version 11.0.

**Table 3:** Bacterial strains, plasmids, and primers used for construction of reporter strains.

Strain, plasmid, or oligonucleotide	Relevant characteristics^1^ or sequences (5′ 3′)	Reference
*Pseudomonas protegens*		
CHA0	Wild type; biocontrol agent; DAPG^+^, PRN^+^, HCN^+^	Stutz et al., 1986
CHA0-*gfp*	CHA0::*att*Tn7-*gfp*; Gm^r^	Péchy-Tarr et al., 2013
*Pseudomonas chlororaphis*		
PCL1391	Wild type; biocontrol agent; HCN^+,^ PHZ^+^	Chin-A-Woeng et al., 1998
PCL1391-*gfp*	PCL1391::*att*Tn7-*gfp*; Gm^r^	This study
*Escherichia coli*		
DH5α	Laboratory strain	Sambrook and Russell, 2001
Plasmids		
pBK-miniTn*7*-*gfp2*	pUC19-based delivery plasmid for miniTn*7-gfp2*; mob^+^ Gm^r^ Cm^r^ Apr^r^	Koch et al., 2001
pME7116	*prnA-gfp* transcriptional fusion; reporter of PRN biosynthetic gene expression in CHA0;Tc^r^	Baehler et al., 2005
pME9010	*mCherry*-based promoter-probe vector derived from pPROBE’-*gfp* (AAV); Km^r^	Rochat et al., 2010
pME9011	*hcnA-mcherry* transcriptional fusion; reporter of HCN biosynthetic gene expression in CHA0; Km^r^	Rochat et al. (2010)
pME9012	*phlA-mcherry* transcriptional fusion; reporter of DAPG biosynthetic gene expression in CHA0; Km^r^	Rochat et al., 2010
pME11011	*prnA-mcherry* transcriptional fusion; reporter of PRN biosynthetic gene expression in CHA0; Km^r^	This study
pME11017	*phzA-mcherry* transcriptional fusion; reporter of PHZ biosynthetic gene expression in PCL1391; Km^r^	This study
pUK21	Cloning vector; Km^r^	Vieira and Messing, 1991
pUX-BF13	Helper plasmid encoding Tn*7* transposition functions; R6K-replicon; Ap^r^	Bao et al., 1991
Oligonucleotides^2^		
P5 BamHI new	CGGGATCCCGGGCTCAAGGACAGTTGGTTCA, BamHI	This study
P6	GGAATTCCCGAGGTACGAAGCGGCCATC, EcoRI	Baehler et al., 2005
phzAF	CGGGATCCCTAACTCCATTTTGAGCACC, BamHI	This study
phzAR	CGAGCTCGCTCAATCTCCAATGAATAAGGGGGCT, SacI	This study

^1^*Abbreviations: Ap^r^, ampicillin; Cm^r^, chloramphenicol; Gm^r^, gentamicin; Km^r^, kanamycin; and Tc^r^, tetracycline resistance, respectively*.

^2^*Restriction sites are underlined*.

In order to tag *P. protegens* CHA0 and *P. chlororaphis* PCL1391 with green fluorescent protein (GFP), a single copy of a *gfp* variant gene constitutively expressed from the *P_tac_* promoter, was inserted into the chromosome using the pBK-miniTn*7*-*gfp2* delivery plasmid (Koch et al., 2001) and the Tn*7* transposition helper plasmid pUX-BF13 (Bao et al., 1991) as described previously (Péchy-Tarr et al., 2013). For use as reporters to monitor the expression of HCN, DAPG, PRN, and PHZ biosynthetic genes, strain CHA0-*gfp* was transformed with pME9011 (*hcnA-mcherry*), pME9012 (*phlA-mcherry*) or pME11011 (*prnA-mcherry*), and strain PCL1391-*gfp* with pME11017 (*phzA-mcherry*) (**Table 3**). To construct the *prnA-mcherry* reporter plasmid pME11011, a 629-bp fragment containing the CHA0 *prnA* promoter was amplified from pME7116 (Baehler et al., 2005) using primers P5 BamHI new and P6 (**Table 3**). The obtained fragment was digested with BamHI and EcoRI and ligated into the *mcherry*-based promoter-probe vector pME9010 (Rochat et al., 2010) opened with the same enzymes. Similarly, for the construction of the *phzA-mcherry* reporter plasmid pME11017, a 961-bp fragment containing the *phzA* promoter (Chin-A-Woeng et al., 2001) was amplified from genomic DNA of PCL1391 (Flury et al., 2016) using primers phzAF and phzAR (**Table 3**). The PCR product was digested with BamHI-SacI and the resulting fragment was first cloned into pUK21 and, from there, into pME9010, both opened with the same restriction enzymes.

### Assay to Monitor Antimicrobial Gene Expression in Field Soils

Antimicrobial gene expression and colonization levels of GFP-marked *P. protegens* CHA0 and *P. chlororaphis* PCL1391 harboring mCherry-based reporter plasmids were monitored on roots of spring wheat grown in the soils sampled at the 10 different Swiss field sites. Untreated seeds of spring wheat cv. Rubli were surface-sterilized for 12 min in 4% NaClO (vol/vol) and then washed with sterile distilled water. Seeds were germinated on soft agar (Agar Agar Serva at 9 g L^-1^; Serva, Heidelberg, Germany) for 48 h at room temperature in the dark. The wheat seedlings were then transferred to 200-mL Erlenmeyer flasks (5-cm opening; Simax, Czech Republic) containing 60 g of soil. In each flask, three seedlings were placed into the soil and inoculated with 1 mL of a suspension of washed cells of the respective *Pseudomonas* reporter strain adjusted within a range from 3.6 10^7^ to 7.8 × 10^7^ cells mL^-1^. Washed cells were prepared from LB cultures grown without antibiotic addition under the conditions described above to an OD_600_ of 0.8 to 1.5, depending on the reporter strain used, whereby choosing a growth stage at which no significant expression of the antimicrobial genes occurred. This was done by determining, with a fluorimetry assay (Baehler et al., 2005), the time point at which the relative red fluorescence emitted by the *Pseudomonas* strains carrying mCherry-based reporter plasmids was not yet significantly different from the red background fluorescence emitted by control strains carrying the empty vector pME9010. Wild-type and GFP-tagged *P. protegens* CHA0 and *P. chlororaphis* PCL1391 (with and without pME9010) were included as control treatments for properly setting green and red fluorescence backgrounds for the FACS-based flow cytometry analysis described below. Flasks were sealed with cotton wool plugs and incubated in a growth chamber set to 60% relative humidity for 16 h with light (176 μE m^-2^ s^-1^) at 25°C, followed by an 8-h dark period at 20°C. After 5 days of incubation, wheat roots from each flask were removed, washed using distilled water to remove loosely adhering soil particles from roots and pooled in 10 mL of autoclaved, ultrapure water contained in a sterile 50-mL Falcon tube. Tubes were agitated for 20 min at 300 rpm in order to remove the majority of bacteria from the roots. The resulting suspensions were filtered using a 5.0-μm sterile syringe single-use filter (Sartorius Stedim Biotech GmbH, Goettingen, Germany), transferred on ice and immediately analyzed by FACS as described below. Dry weights of wheat roots were recorded and the number of GFP-marked *Pseudomonas* cells present in the root washes were determined by FACS and recorded as cells g^-1^ of root.

### FACS Analysis

Green fluorescent protein and mCherry expression levels in *Pseudomonas* reporter cells in natural soils were quantified with a Becton–Dickinson LSRFortessa flow cytometer. Size and granularity of *Pseudomonas* cells and particles were determined by measuring the forward scatter (FSC-A) and side scatter (SSC-A) signals, respectively. FSC-A signals were collected with a photodiode detector (set to 350 V), in the range of 483 to 493 nm (488/10 BP filter), with a threshold set to 200. SSC-A signals were detected with a photomultiplier tube (PMT) (detector G, set to 300 V) in the range of 483 to 493 nm (488/10 BP filter). Green fluorescence signals were collected with the PMT detector E (set at the voltage of 676 V), in the range of 515 to 545 nm (530/30 BP filter, 505 LP mirror). Red fluorescence signals were detected using the PMT detector C (set to 700 V), between 600 and 620 nm (610/20 BP filter, 600 LP mirror). For FACS analysis, aliquots of 300 μL of filtered root-washes were placed into Nunc MaxiSorp flat-bottom 96-well plates (Sigma–Aldrich, Buchs, Switzerland), and each sample was mixed three times. The analyzed volume was standardized to 200 μL, allowing the detection of 500–10,000 GFP events, depending on the analyzed soil. Gating of GFP-marked bacteria was done by delimiting on the FSC-A/FITC-A density plot particles with green fluorescence values above the background fluorescence noise (i.e., autofluorescence emitted by root and soil particles, cell fragments or bacterial cells not expressing GFP). Control samples obtained from soils amended with pure water, wild-type *P. protegens* CHA0 and *P. chlororaphis* PCL1391, and GFP-tagged strains CHA0-*gfp* and PCL1391-*gfp* were used to identify the GFP background fluorescence in soil extraction samples. The red fluorescence emitted by the gated GFP-tagged *Pseudomonas* cells was then analyzed on the PE-Texas Red-A histogram and on the FSC-A/PE-Texas Red-A density plot, allowing to detect and analyze all GFP-marked cells actively expressing their mCherry-based reporter fusion. Control samples including CHA0-*gfp* and PCL1391-*gfp* without mCherry-based vector, or carrying the empty pME9010 vector were used to define the mCherry background fluorescence among the GFP-marked *Pseudomonas* population. The median of red fluorescence emitted by the *Pseudomonas* cells was calculated using the BD FACSDiva software version 8.0 (Becton–Dickinson). To determine root colonization levels of *Pseudomonas* reporter strains, the GFP tag was used to count by FACS the number of reporter cells present in the analyzed 200 μL of filtered root-wash and then to calculate their concentration per gram of dry roots.

### Data Analysis

Statistical data analysis was carried out with the open source software R version 3.2.3 (R Core Team, 2015). Shoot fresh weights of cucumber and wheat plants obtained from the Pu and Gt infection assays, respectively, were analyzed as a proxy for soil disease resistance. Shoot weights from samples where pathogen inoculum was added were normalized against the shoot weights from non-infested control plants grown in the same soil, to minimize variation due to different nutrient contents in the different soils. Data were checked for normal distribution with the Shapiro–Wilk test and by plotting QQ-Plots. Equality of variance was verified with Bartlett’s test. Analysis of variance was carried out with a non-parametric test (Kruskal–Wallis test, significance level *p* < 0.05), followed by a *post hoc* test (kruskalmc, R package ‘Pgirmess’).

The abundance of *phlD*, *phzF*, and *prnD* harboring bacterial cells on wheat roots was calculated with the *in vivo* standard curves described above. Efficiencies and detection limits of qPCR assays determined by *in vivo* standard curves are indicated in Supplementary Table S2. Cycle threshold values obtained from the *in vivo* standard curves and from the samples were normalized for differences in DNA extractions as described by Von Felten et al. (2010). Normalized values were used for further analysis. The average values obtained from the three technical replicates of each qPCR assay were used for statistical analysis, which was performed as described above for shoot weights.

Data on the expression of reporters of antimicrobial genes in the different soils represent the medians of three independent repetitions of the same experiment, with nine replicates per treatment in each experiment. Significant differences between treatments were calculated with a non-parametric Kruskal–Wallis test (significance level *p* < 0.05), followed by Dunn’s test for *post hoc* comparisons.

Data used for the heat map showing rankings of pathogen suppression, abundance of antimicrobial genes, and expression of antimicrobial genes by reporter strains in the different soils were normalized using the function ‘scale’ (R package ‘stats’). Correlations between pathogen suppression, gene abundance, gene expression and abiotic soil parameters were inferred with Spearman’s rho rank correlation (significance level *p* < 0.05). Data were displayed in a heat map with the functions ‘levelplot’ (R package ‘lattice’) or ‘corrplot’ (R package ‘corrplot’).

## Results

### Resistance of Swiss Agricultural Soils to Soilborne Pathogens

**FIGURE 2:**
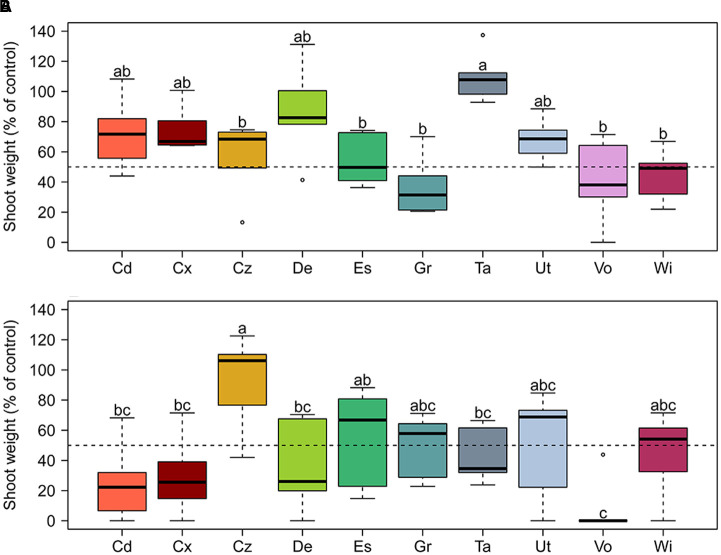
**Relative resistance of 10 representative Swiss agricultural soils with a cereal-oriented cropping history to the soil-borne pathogens (A)** *Gaeumannomyces tritici* (Gt) and **(B)** *Pythium ultimum* (Pu). Increasing concentrations of pathogen inoculum were added to the soil before planting with spring wheat (Gt experiment) or cucumber (Pu experiment) seedlings. Data shown here are for Gt at 0.6 g and Pu at 0.125 g inoculum per pot. Results for the other inoculum concentrations tested are shown in Supplementary Figures S1, S2. Each pathogen concentration and soil was tested in six replicate pots. Soil resistance is shown as fresh shoot weight of plants in artificially pathogen-infested soil compared to fresh shoot weight of control plants grown in non-infested soil. The dotted line indicates 50% of shoot weight compared to the control. Letters indicate significant differences (Kruskal–Wallis test, *p* < 0.05). Sampling sites: Cd, Cadenazzo; Cx, Courtedoux; Cz, Cazis; De, Delley; Es, Eschikon; Gr, Grangeneuve; Ta, Taenikon; Ut, Utzenstorf; Vo, Vouvry; Wi, Witzwil.

The general resistance of 10 Swiss agricultural soils (**Figure 1** and **Table 1**) to soilborne pathogens was tested in a greenhouse assay in which increasing quantities of Gt or Pu inoculum were added to soil samples planted with spring wheat or cucumber, respectively. Resistance to both pathogens varied between soils. In the Gt-infested soils at 0.6 g inoculum per pot, shoot fresh weights of wheat plants ranged from 31% in Grangeneuve soil to 107% in Taenikon soil of the weights of control plants grown in not artificially infested soils (**Figure 2A**). The shoot fresh weights of wheat grown in infested Taenikon soil were significantly higher (1.3–3.5-fold) than those of plants grown in infested soils from Cazis, Eschikon, Grangeneuve, Vouvry, and Witzwil. Similar trends were observed at other inoculum quantities (Supplementary Figure S1). Plants grown in Taenikon soil had the highest shoot fresh weights at all Gt concentrations used except the highest (6 g of inoculum per pot), at which the fungal pathogen heavily affected plant growth in all the soils and reduced shoot weights by 60–90% (Supplementary Figure S1D).

When the soils were artificially infested with Pu inoculum at 0.125 g per pot, median shoot fresh weights of cucumber plants ranged from 0% in Vouvry soil to 106% in Cazis soil of those of control plants grown in non-infested soils (**Figure 2B**). The shoot fresh weights of cucumber grown in Pu-infested Cazis soil were significantly higher (three–fivefold) than those of plants grown in infested soils from Cadenazzo, Courtedoux, Delley, Taenikon, and Vouvry (**Figure 2B**) and similar trends were observed for other inoculum densities (Supplementary Figure S2). Plants grown in soil from the Cazis field site had the highest shoot weights at all Pu concentrations, while plants grown in soil from the Vouvry field had the lowest shoot weights at all levels of the pathogenic oomycete except at 0.25 g per pot.

Individual soils did not display equal resistance levels to the two soilborne pathogens. While the soils from the Taenikon and Delley field sites were the most resistant against Gt, they were among the least resistant against Pu (**Figure 2** and Supplementary Figures S1, S2). Likewise, the soil from Cazis was the most resistant against Pu, but was only moderately resistant against Gt.

To account for potential effects of resident Gt and Pu populations on the outcome of the soil resistance experiments, a qPCR method targeting the ITS rRNA gene region was used to detect and quantify the two pathogens in the rhizoplane of spring wheat plants grown in the not artificially infested control treatments of all soils. Gt could not be detected in any of the samples. By contrast, Pu was detected on the plant roots in all ten soils, but there was no significant difference in the abundance of the oomycete pathogen among the individual soils (Supplementary Figure S3).

In summary, the 10 agricultural soils strongly varied in their resistance against soilborne pathogens. However, the soil resistance levels observed for the two investigated pathogens in general were different; i.e., some soils displaying high resistance to Pu were highly susceptible to Gt and vice versa, pointing to specificity in the buffering capacity of individual soils toward specific soilborne pathogens.

### Abundance of *phlD*^+^ Pseudomonads, *phzF*^+^ Pseudomonads, and *prnD*^+^ Bacteria on Roots of Wheat Grown in Swiss Agricultural Soils

The abundance of bacterial cells harboring *phlD, phzF*, and *prnD* required for the biosynthesis of the antimicrobials DAPG, PHZ, and PRN, respectively, was quantified by qPCR on roots of spring wheat grown in the 10 Swiss agricultural soils. As detailed in section “Materials and Methods,” we assume that *phlD*^+^ and *phzF*^+^ cells quantified in our assays correspond to cell numbers of DAPG and PHZ producing pseudomonads, whereas *prnD*^+^ cells correspond to cell numbers of PRN producing bacteria. Since the investigated genes are present as one copy per bacterial cell, we also refer to the abundance of cells harboring an antimicrobial gene as gene abundance.

**FIGURE 3:**
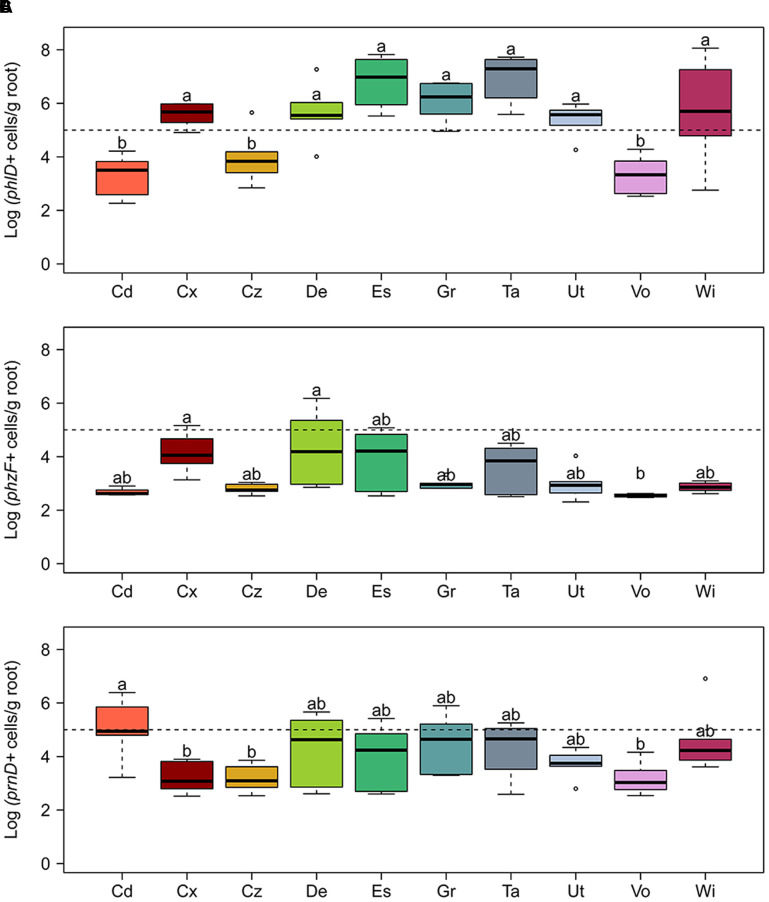
**Abundance of bacterial cells harboring genes required for the biosynthesis of the antimicrobial compounds (A)** 2,4-diacetylphloroglucinol (*phlD*), **(B)** phenazine (*phzF*), and **(C)** pyrrolnitrin (*prnD*) on roots of spring wheat in 10 Swiss agricultural soils. Cells harboring the antimicrobial genes were quantified by qPCR. The dotted line indicates 10^5^ cells per gram of root dry weight. For each soil, six replicates were used. Letters indicate significant differences (Kruskal–Wallis test, *p* < 0.05). Sampling sites: Cd, Cadenazzo; Cx, Courtedoux; Cz, Cazis; De, Delley; Es, Eschikon; Gr, Grangeneuve; Ta, Taenikon; Ut, Utzenstorf; Vo, Vouvry; Wi, Witzwil.

The abundance of the *phlD*^+^ pseudomonads in all studied soils in general was higher than the abundance of the *phzF*^+^ and *prnD*^+^ bacteria, and in some soils reached 10^7^ cells per gram of root dry weight, whereas the abundance of the latter remained below 10^6^ gene copies per gram of root dry weight (**Figure 3**). For individual genes, pronounced differences in abundance between soils were observed. The biggest differences were found for *phlD*^+^ cells with approximately 10^4^-fold higher numbers on roots of wheat grown in soil from Taenikon compared to those grown in soil from Vouvry (**Figure 3A**). The abundance of *phlD*^+^ pseudomonads was significantly higher in soils from Courtedoux, Delley, Eschikon, Grangeneuve, Taenikon, Utzenstorf, and Witzwil than in soils from Cadenazzo, Cazis, and Vouvry. The abundances of *phzF*^+^ pseudomonads and *prnD*^+^ bacteria varied at maximum 100-fold between the different soils. The number of pseudomonads harboring the *phzF* gene was significantly higher on roots samples from Courtedoux and Delley soils compared to samples extracted from Vouvry soil (**Figure 3B**). The abundance of *prnD*^+^ bacteria was significantly higher in soil samples from Cadenazzo, compared to those from Courtedoux, Cazis, and Vouvry (**Figure 3C**). Taken together, for all three investigated antimicrobial genes pronounced differences in the abundances were found between the individual agricultural soils, indicating that the different soils may sustain to different extents specific populations of pseudomonads producing DAPG, PRN, and/or PHZ.

### Expression of Antimicrobial Genes in Swiss Agricultural Soils

The relative capacity of the 10 different Swiss agricultural soils to sustain the expression of biosynthetic genes for the antimicrobial compounds DAPG, HCN, PRN, and PHZ on roots was followed using GFP-tagged *Pseudomonas* strains carrying mCherry-based reporter plasmids inoculated into soil microcosms planted with spring wheat. *P. protegens* CHA0-*gfp* carrying plasmid pME9012 (*phlA*-*mcherry*), pME9011 (*hcnA-mcherry*), or pME11011 (*prnA*-*mcherry*), or *P. chlororaphis* PCL1391-*gfp* carrying pME11017 (*phzA*-*mcherry*) served as reporter strains. GFP fluorescence (identifying the tagged reporter strains) and relative mCherry fluorescence intensities (reporting expression levels of respective antimicrobial genes) of cells in root washes extracted from the different soils were recorded with FACS-based flow cytometry. Data presented as total antimicrobial gene expression by all cells of the respective reporter strain per gram dry weight of roots, and they were calculated by multiplying the median gene expression per cell with the total number of reporter cells per gram dry weight of roots (Supplementary Table S3).

**FIGURE 4:**
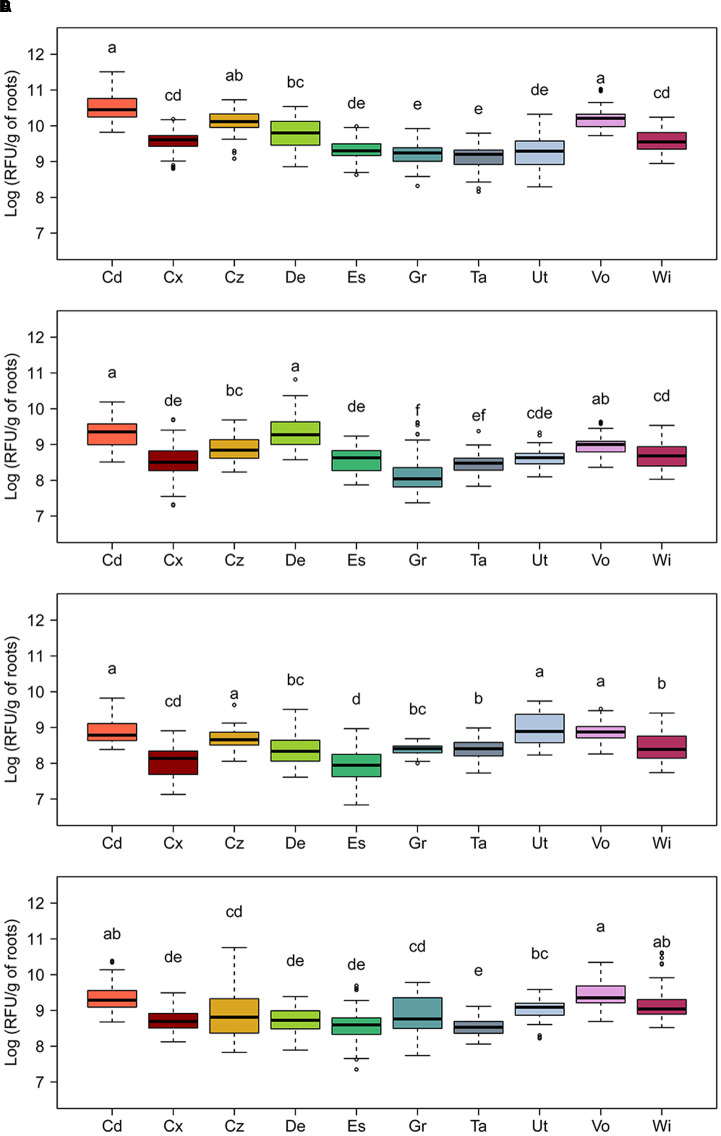
**Relative expression of genes required for the biosynthesis of the antimicrobial compounds (A)** 2,4-diacetylphloroglucinol (*phlA*), **(B)** hydrogen cyanide (*hcnA*), **(C)** pyrrolnitrin (*prnA*), and **(D)** phenazines (*phzA*) on roots of spring wheat in 10 Swiss agricultural soils. Expression was monitored by fluorescence-activated cell-sorting-based flow cytometry using GFP-tagged strains of *Pseudomonas protegens* (CHA0-*gfp*) carrying reporter plasmids pME9012 (*phlA-mcherry*), pME9011 (*hcnA-mcherry*), or pME11011 (*prnA-mcherry*) or of *P. chlororaphis* (PCL1391-*gfp*) carrying reporter plasmid pME11017 (*phzA*-*mcherry*). Seedlings inoculated with the reporter strains were grown in soil microcosms for 5 days prior to analysis of bacterial cells in root washes. Data are shown as relative fluorescence units (RFU) per gram of root dry weight, and were calculated as the median mCherry expression per GFP-tagged *Pseudomonas* cell multiplied with the total number of GFP-tagged *Pseudomonas* cells per gram of root. Results from three independent experiments with nine replicates each are presented. Since Kruskal–Wallis analyses did not reveal significant experiment × treatment interactions, data of the three experiments were pooled for statistical analysis. Letters indicate significant differences (Dunn test, *p* < 0.05). Sampling sites: Cd, Cadenazzo; Cx, Courtedoux; Cz, Cazis; De, Delley; Es, Eschikon; Gr, Grangeneuve; Ta, Taenikon; Ut, Utzenstorf; Vo, Vouvry; Wi, Witzwil.

The levels of total expression of all investigated antimicrobial genes varied significantly among the 10 field soils, with the highest variations observed for DAPG and HCN biosynthetic genes (**Figure 4**). The soil from Cadenazzo supported the highest levels of total *phlA* expression on the roots, which were approximately 16-fold higher than those measured on roots growing in soil from Taenikon, which yielded the lowest expression levels (**Figure 4A**). The Cadenazzo soil also supported highest levels of total *hcnA* expression among all 10 soils, and these levels were approximately 20-fold higher than *hcnA* expression levels recorded in the soil from the Grangeneuve field site, which was the least favorable to the expression of this antimicrobial gene (**Figure 4B**). Levels of total expression of PRN and PHZ biosynthetic genes appeared to be less variable among the 10 soils. In particular, *prnA* expression levels were eightfold higher in the soil from Utzenstorf that supported the highest expression compared to the soil from Eschikon that supported the lowest expression (**Figure 4C**). Levels of overall *phzA* expression varied only about fivefold between the most contrasting soils from the Cadenazzo and Taenikon field sites, respectively (**Figure 4D**). Several individual soils sustained total expression levels for all investigated antimicrobial genes to a similar extent. This was evident notably for soils from Cadenazzo and Vouvry in which all four antimicrobial genes attained high total expression levels, while soils from Eschikon, Grangeneuve and Taenikon supported significantly lower overall expression levels of these genes (**Figure 4**).

**FIGURE 5:**
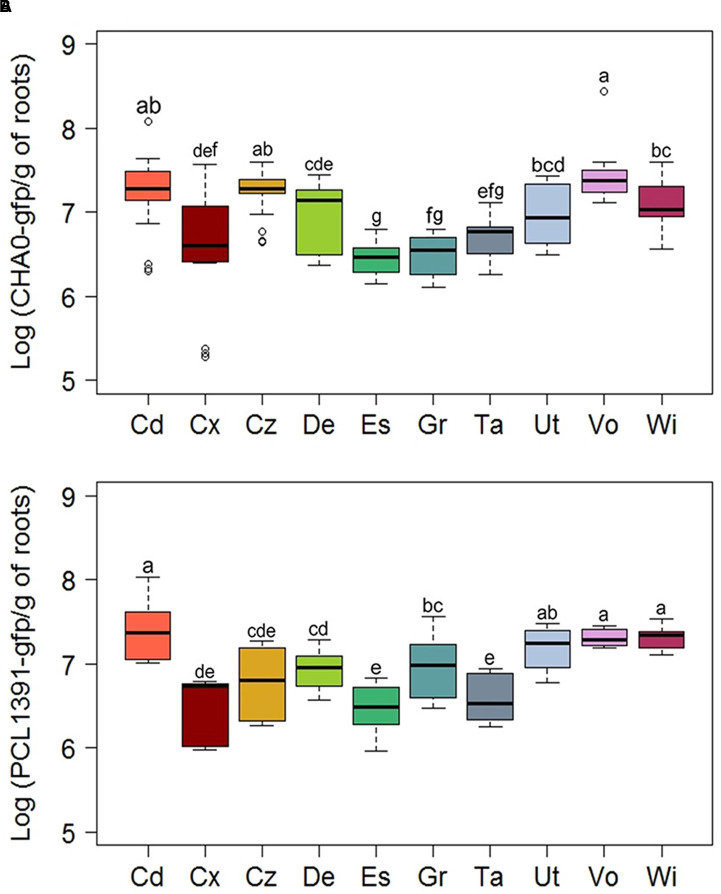
**Root colonization levels of *Pseudomonas* reporter strains in 10 Swiss agricultural soils planted with spring wheat**. Cells of GFP-tagged strains of **(A)** *P. protegens* (CHA0-*gfp*) or **(B)** *P. chlororaphis* (PCL1391-*gfp*) were monitored by fluorescence-activated cell-sorting-based flow cytometry. Data show numbers of GFP-tagged cells per gram of root dry weight. Results from three independent experiments with nine replicates each are presented. Since Kruskal–Wallis analyses did not reveal significant experiment × treatment interactions, data of the three experiments were pooled for statistical analysis. Letters indicate significant differences (Dunn test, *p* < 0.05). Sampling sites: Cd, Cadenazzo; Cx, Courtedoux; Cz, Cazis; De, Delley; Es, Eschikon; Gr, Grangeneuve; Ta, Taenikon; Ut, Utzenstorf; Vo, Vouvry; Wi, Witzwil.

The Cadenazzo and Vouvry soils appeared to favor also highest levels of root colonization (1.9 × 10^7^ to 2.4 × 10^7^ cells g^-1^ of dry root weight) by *P. protegens* and *P. chlororaphis* among the 10 soils tested, while the soils from Eschikon, Grangeneuve, and Taenikon were among those supporting lower levels of root colonization (2.9 × 10^6^ to 9.7 × 10^6^ cells g^-1^ of dry root weight) (**Figure 5** and Supplementary Table S3). However, whereas the soil from Cadenazzo was indeed also the field soil supporting highest *phlA*, *hcnA*, *prnA*, and *phzA* expression levels in individual reporter cells, the median single cell expression of these genes was significantly lower in the soil from Vouvry (Supplementary Table S3). For the Vouvry soil, it thus seems that the high total expression in the reporter population on the roots was mainly due to the higher colonization levels attained in this soil. Likewise, the relatively high single cell expression but low colonization levels in the Eschikon and Taenikon soils (**Figure 5** and Supplementary Table S3) resulted in the significantly lower overall expression as compared to the Cadenazzo and Vouvry soils (**Figure 5**). By contrast, in the soil from Grangeneuve, single cell expression levels of antimicrobial genes as well as colonization levels were consistently relatively low (Supplementary Table S3). Similar overall gene expression levels may therefore reflect contrasting levels of single cell gene expression and colonization in individual soils.

In summary, the expression of the investigated antimicrobial genes strongly varied between the studied agricultural soils. Some soils seem to favor higher levels of overall antimicrobial gene expression whereas others apparently support the expression of these genes much less. The findings suggest that the expression of different antimicrobial genes may be induced by the same soil factors (or similar combinations thereof).

### Relationships between Pathogen Resistance and Abundance and Expression of Antimicrobial Genes in Swiss Agricultural Soils

**FIGURE 6:**
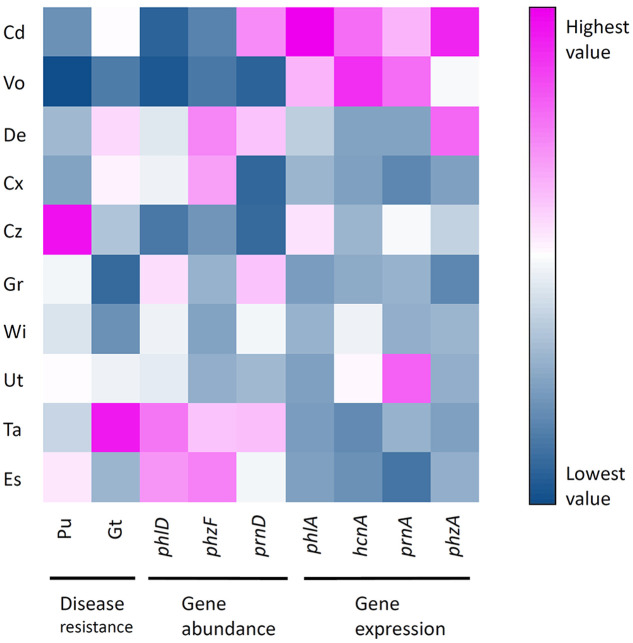
**Heatmap showing normalized-values of disease resistance, antimicrobial gene abundance and antimicrobial gene expression measured in 10 representative Swiss agricultural soils with a cereal-oriented cropping history**. The color scale depicts highest (fuchsia) via intermediate (white) to lowest (blue) values for each variable. Sampling sites: Cd, Cadenazzo; Cx, Courtedoux; Cz, Cazis; De, Delley; Es, Eschikon; Gr, Grangeneuve; Ta, Taenikon; Ut, Utzenstorf; Vo, Vouvry; Wi, Witzwil.

Results obtained from experiments on pathogen resistance of soils, abundance and expression of antimicrobial genes were displayed in a gradient map (**Figure 6**). Soils were grouped in three clusters. The first cluster consisted of soils supporting high abundances but low expression levels of antimicrobial genes (i.e., soils from Eschikon and Taenikon field sites). The second cluster consisted of soils supporting high antimicrobial gene expression levels but low abundances of bacteria harboring antimicrobial genes (i.e., soils from Cadenazzo and Vouvry field sites). The third cluster consisted of soils ranging in between the two other clusters. Two soils displayed high resistance against soilborne plant pathogens, i.e., the soil from Taenikon against Gt and the soil from Cazis against Pu. Although the soil from Taenikon was the one supporting the highest abundance of pseudomonads harboring the DAPG biosynthetic gene *phlD*, resistance to the soilborne pathogens did not generally cluster with high abundances or expression levels of antimicrobial genes (**Figure 6**).

**FIGURE 7:**
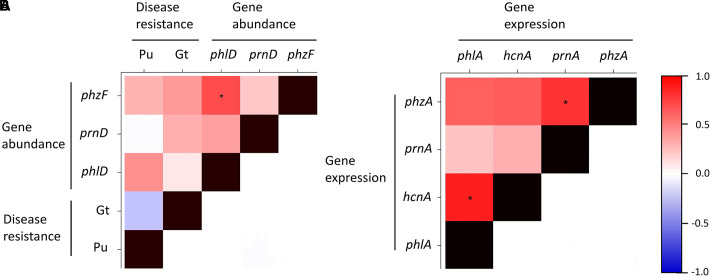
**Heatmap showing Spearman’s rank correlations for disease resistance and abundance of antimicrobial genes (A)** and for expression levels of antimicrobial genes **(B)** in 10 representative Swiss agricultural soils with a cereal-oriented cropping history. The abundance of antimicrobial genes is defined as the number of bacterial cells harboring the indicated gene. Significant correlations (*p* < 0.05) are highlighted with asterisks. The color scale to the right of the matrix indicates rho correlation coefficients.

Correlation analysis revealed that the expression of the four studied antimicrobial genes was positively correlated (**Figure 7B**). A similar trend could be observed for the abundance of the antimicrobial genes (**Figure 7A**). Resistance to soilborne pathogens was not significantly correlated to the abundance of antimicrobial genes, although a weak positive correlation could be observed between the abundance of bacteria harboring individual antimicrobial genes and resistance to pathogens (**Figure 7A**). These results suggest that the expression of the investigated antimicrobial genes is similarly influenced by biotic and abiotic factors prevailing in the respective soils. The same could be true for the abundance of bacteria harboring these antimicrobial genes. Our results further indicate that the abundance of such bacteria probably plays only a limited role in the resistance of the investigated cereal crop-oriented agricultural soils to the soilborne pathogens Pu and Gt.

### Relationships between Soil Parameters, Pathogen Resistance, Abundance and Expression of Antimicrobial Genes

**FIGURE 8:**
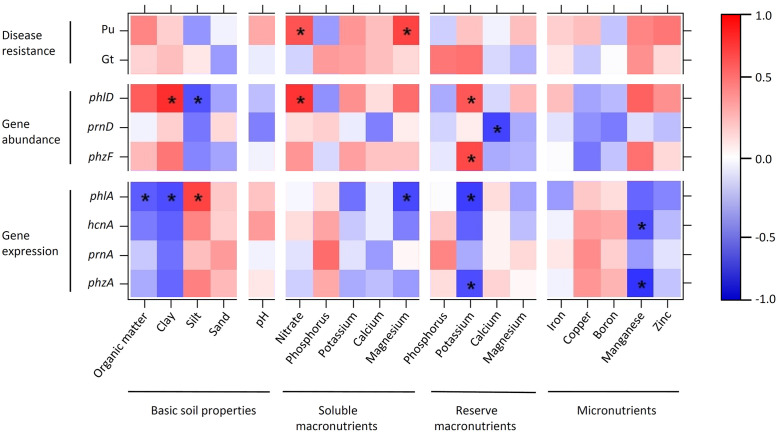
**Heatmap showing Spearman’s rank correlations between soil parameters, disease resistance, and abundance and expression of antimicrobial genes in 10 representative Swiss agricultural soils with a cereal-oriented cropping history**. The abundance of antimicrobial genes is defined as the number of bacterial cells harboring the indicated gene. Significant correlations (*p* < 0.05) are highlighted with asterisks. The color scale to the right of the matrix indicates rho correlation coefficients.

The physical and chemical properties of the 10 Swiss agricultural soils investigated in this study were analyzed (**Figure 1** and **Table 1**) and correlated with their resistance to pathogens, the abundance of antimicrobial genes and the expression of antimicrobial genes (**Figure 8**). Macronutrients were extracted from soils with the water or ammonium acetate-EDTA (AA-EDTA) soil extraction procedures routinely used in Swiss agriculture to account for, respectively, soluble, i.e., readily plant-available macronutrients and bound, reserve macronutrients that become available to plants at mid or long term (Agroscope, 2006; Stünzi, 2007). Plant micronutrients were extracted with AA-EDTA.

Organic carbon and clay, and silt and sand inversely influenced antimicrobial gene abundance and expression. Abundance of DAPG biosynthesis genes (recorded for *phlD*) was significantly positively correlated with clay and significantly negatively correlated with silt, while expression of these genes (recorded for *phlA*) was significantly negatively correlated with organic carbon and clay and significantly positively correlated with silt (**Figure 8**). Similar trends were also observed for the pyrrolnitrin and phenazine biosynthetic genes *prnA* and *phzF*, respectively. No clear positive or negative correlation was found between pH and antimicrobial gene abundance or expression (**Figure 8**). Nitrate concentration in soil was positively correlated with the abundance of bacteria harboring the investigated antimicrobial genes; in particular, it was significantly positively correlated with the abundance of *phlD*^+^ pseudomonads (**Figure 8**). Antimicrobial gene expression was neither clearly positively nor clearly negatively correlated with nitrate. Of the other macronutrients, potassium inversely influenced abundance and expression of antimicrobial genes. Reserve potassium extracted with AA-EDTA was significantly positively correlated with the abundance of *phlD*^+^ and *phzF*^+^ pseudomonads and significantly negatively with *phlA* and *phzA* expression (**Figure 8**). A similar trend was also observed for water-extracted, i.e., readily plant available magnesium. Among the measured micronutrients, a significant effect was only observed for manganese, which was significantly negatively correlated with *phzA* and *hcnA* expression (**Figure 8**).

Resistance to soilborne pathogens was not significantly correlated to any soil factor in the case of Gt, and significantly positively correlated to nitrate and magnesium extracted with water in the case of Pu (**Figure 8**). The resistance to both pathogens was positively, but not significantly correlated to organic carbon, clay, potassium, manganese, and zinc.

Taken together, these analyses indicate that soil physical and chemical properties have contrasting and subtle effects on the abundance and expression of antimicrobial genes. No pronounced correlations between soil properties and general disease resistance of soils could be observed for the 10 agricultural field sites investigated. Remarkably, all soil factors that were positively correlated with the abundance of *phlD*^+^ and *phzF*^+^ pseudomonads were also positively correlated with Pu resistance.

## Discussion

Resistance to soilborne diseases and beneficial microbial populations involved have been studied extensively in soils specifically suppressive to one particular pathogen species (Weller et al., 2002; Mendes et al., 2011; Raaijmakers and Mazzola, 2016). However, virtually nothing is known about interactions between soilborne pathogens and beneficial microorganisms in common agricultural soils, i.e., in soils that lack specific disease suppressiveness. Similarly, it is not clear whether a particular soil may simultaneously exhibit suppressiveness toward multiple soilborne pathogens. To our best knowledge, the present study is the first to compare side-by-side a range of common agricultural soils for their resistance toward two soilborne pathogens, *P. ultimum* (Pu) and *G. tritici* (Gt), as well as for the abundance and expression of biosynthetic genes required for the production of antimicrobial compounds by plant-beneficial pseudomonads.

First, we investigated the capacity of different Swiss agricultural soils to buffer the attack of two soilborne pathogens by testing the growth of crop plants in these soils after amendment with increasing quantities of pathogens up to very high concentrations. Individual soils differed markedly in their respective resistance to the two pathogens. Several soils that showed comparatively high suppressiveness toward Pu were poorly or only moderately resistant to Gt and vice versa. These results indicate that conventional agricultural soils do not necessarily exhibit a general level of resistance toward a range of soilborne pathogens, but rather display variable resistance levels toward specific pathogens, which are likely modulated by different microbial and abiotic soil factors. Nevertheless, soils suppressive to more than one pathogen have been reported, notably the *Fusarium* wilt of pea suppressive soils in Mt. Vernon, WA, USA (Landa et al., 2002; Weller et al., 2007), which are also suppressive to take-all of wheat (Allende Molar, 2006; Allende-Molar and Weller, personal communication).

Soil resistance to soilborne pathogens has often been linked to the abundance of pseudomonads producing antimicrobial compounds (Stutz et al., 1986; Raaijmakers et al., 1997; Weller et al., 2002, 2007; Haas and Défago, 2005; Raaijmakers et al., 2008; Mazurier et al., 2009; Almario et al., 2014). For this reason, we have used a qPCR approach targeting biosynthetic genes for DAPG, PHZ, and PRN in bacterial cells present on roots of wheat grown in the agricultural soils that we tested for disease resistance. While the qPCR assays used in this study to quantify *phlD*^+^ and *phzF*^+^ bacteria are specific for fluorescent pseudomonads (see Supplementary Table S1), the assay used to quantify *prnD*^+^ cells additionally detects *Burkholderia* and *Serratia* (Garbeva et al., 2004). However, 16S rRNA amplicon sequencing performed on root samples from wheat grown in the same soils (Dennert et al., unpublished results) showed that the relative abundance of *Pseudomonas* (4.4–25.2%) was markedly higher than the relative abundances of *Burkholderia* (0.003–0.53%) and *Serratia* (0.003–1.21%). Therefore, we assume that the *prnD* genes detected in the present study predominantly derive from pseudomonads. Most studies that quantified pseudomonads harboring antimicrobial genes so far were carried out using cultivation-dependent approaches, e.g., colony plating/colony hybridization assays or endpoint dilution assays followed by PCR (Raaijmakers et al., 1997; Meyer et al., 2010; Mavrodi D.V. et al., 2012; Mavrodi O.V. et al., 2012). An examination of genome sequences published by Flury et al. (2016) and other investigators indicated that fluorescent pseudomonads harbor only one copy of *phlD*, *phzF*, or *prnD* per cell. Accordingly, we did not find any significant difference between the abundances of *phlD* and *phzF* quantified by qPCR or a cultivation-dependent endpoint dilution assay in samples from Taenikon (Supplementary Figure S4). Still, qPCR assays can potentially detect viable but non-culturable or even dead cells, thus caution is required when comparing our findings with results of cultivation-dependent experiments.

Studies simultaneously investigating the abundance of several antimicrobial genes in soil are rare. Raaijmakers et al. (1997) used a colony-hybridization assay to quantify pseudomonads harboring DAPG or PHZ genes in different take-all suppressive and conducive US soils. They found pseudomonads harboring DAPG genes to be enriched in suppressive soils, but much less abundant or below the detection limit in conducive soils and they did not detect pseudomonads harboring PHZ genes in any of the tested soils. In our study, we found *phzF*^+^ pseudomonads to be present in all investigated agricultural soils. However, in general their abundance was quite low, ranging from 2.5 to 5.3 log cells/g root, compared to another US study on soils of the Columbia Plateau of the Pacific Northwest, in which the abundance of PHZ-producing pseudomonads detected by endpoint-dilution assays coupled with *phzF*-specific PCR was up to 100-fold higher in certain soils (Mavrodi D.V. et al., 2012; Mavrodi O.V. et al., 2012). It has been suggested that PHZ producing pseudomonads are more abundant in dryland fields without irrigation, where yearly rainfall ranges from 150 to 300 mm, compared to irrigated fields (Mavrodi O.V. et al., 2012). At the sampling sites of the present study, average annual rainfall was high, ranging from 610 mm per year to 1780 mm per year, which could be a possible reason for the rather low abundance of *phzF*^+^ pseudomonads detected here.

The abundance of DAPG producing pseudomonads in the investigated soils varied strongly, from 2.2 to 8.0 log cells/g root. We detected higher but also lower numbers compared to previous studies, which may be explained by the very different types of soils we investigated (**Table 1**). Two studies using terminal endpoint dilution assays followed by *phlD*-specific PCR, one by Meyer et al. (2010) on two Swiss agricultural soils and one by Mavrodi O.V. et al. (2012) on irrigated fields of the Pacific Northwest, detected *phlD*^+^ pseudomonads at levels ranging from 4.5 to 6.5 log CFU/g on roots of wheat. The population levels of pseudomonads on roots of wheat grown in the Delley soil reported by Meyer et al. (2010) were similar to the numbers of *phlD*^+^ pseudomonads detected by qPCR in our study. Bacteria harboring *prnD* were detected in the 10 Swiss soils investigated here at abundances comparable to those found in different types of agricultural soils in a previous study (Garbeva et al., 2004).

The abundance of antimicrobial genes, respectively, of the bacteria harboring these genes, does only reflect the bacterial population that potentially could produce a particular antimicrobial compound but is not useful to identify the conditions that favor the proliferation and the consequent niche domination for a given bacterial species, as well as the production of specific antimicrobials *in situ*. The present study is the first to monitor side-by-side the expression of several biosynthetic genes for antimicrobial compounds in different agricultural soils, i.e., in particular *phlA*, *hcnA*, *prnA*, and *phzA*, reflecting the biosynthesis of DAPG, HCN, pyrrolnitrin, and phenazines (Baehler et al., 2005; Chin-A-Woeng et al., 2005; Rochat et al., 2010). This was done by FACS-based monitoring of dual-labeled *Pseudomonas* reporter strains that carry a GFP cell tag and a mCherry-based reporter allowing to record the relative expression of a specific antimicrobial gene in individual cells of a *Pseudomonas* population. A similar technique was used by de Werra et al. (2008) and Rochat et al. (2010) for measuring the expression of *phlA*, *hcnA*, and *prnA* genes by *Pseudomonas* reporter strains on roots of different plant varieties in soilless systems. However, this is the first time that the combination of FACS and fluorescent reporter strains was applied to measure antimicrobial gene expression on plant roots grown in different natural soils. Moreover, to our best knowledge, there were no reports so far on phenazine gene expression in soil or on plant roots. To date, only a handful of studies attempted to determine the expression of antimicrobial genes of pseudomonads in soil (DeCoste et al., 2011; Novinscak and Filion, 2011; Almario et al., 2013b), mainly because of practical challenges. In our study, our initial attempts of tracking fluorescence of reporter strains extracted from roots and soil with microscopy revealed impracticable because of the strong autofluorescence of the investigated soil and root material. To address this problem, we modified the FACS approach of Rochat et al. (2010) and used GFP as a constitutively expressed cell marker and mCherry-based reporters for monitoring antimicrobial gene expression. This approach allowed to specifically track the *Pseudomonas* reporter cells and their expression of select antimicrobial genes in natural soil. However, due to the lack of suitable fluorescent proteins allowing reliable visualization of bacteria cells and gene expression in natural soil, a major limitation of this method is that it is impossible to monitor the expression of two or more antimicrobial genes in the same bacterial cell simultaneously. An alternative method of measuring gene expression in soils involves the extraction of total RNA followed by quantitative reverse transcription PCR of bacterial transcripts of interest. This method was used to quantify the expression of DAPG and HCN biosynthetic genes in natural and artificial soils (DeCoste et al., 2011; Novinscak and Filion, 2011; Almario et al., 2013b). However, the main problem with this approach is the inefficiency of RNA extraction from a complex material such as soil along with the limited abundance of transcripts for antimicrobial genes.

We observed an interesting trend where in soils from Cadenazzo and Vouvry the expression of the four studied genes was markedly enhanced, while lower levels of gene expression occurred in soils from Eschikon, Grangeneuve, and Taenikon. The observation that a specific group of soils is capable of promoting or hampering the expression of four distinct antimicrobial genes in pseudomonads suggests that something in the abiotic or biotic composition of these soils can modulate the production of corresponding plant-beneficial compounds in the *P. protegens* and *P. chlororaphis* reporter strains. Furthermore, we observed that the soil type also influenced the capacity of both pseudomonads to colonize wheat, e.g., plants grown in soils from Cadenazzo and Vouvry supported higher populations than those grown in the Eschikon soil (**Figure 5**). However, the differences in root colonization by the two pseudomonads do not explain entirely the difference in gene expression observed in the different soils (**Figures 4**, **5** and Supplementary Table S3).

The relative importance of the abundance of *Pseudomonas* spp. for suppression of soilborne pathogens was questioned recently (Kyselkova et al., 2014). Indeed, DAPG and HCN producing pseudomonads could be isolated not only from suppressive but also from conducive soils, leading to the hypothesis that differential environmental factors prevailing in the two types of soils may shape the expression of relevant biocontrol genes in *Pseudomonas* bacteria and thus disease suppression (Ramette et al., 2006). Moreover, the abundance of the DAPG biosynthesis gene *phlD* was not indicative of the resistance of soils to black root rot of tobacco (Almario et al., 2013b; Kyselkova et al., 2014). Our results support the hypothesis that the abundance of pseudomonads producing antimicrobial metabolites may, in fact, be less important for disease suppression in the rhizosphere than previously hypothesized, at least for certain soils. We failed to detect any significant positive correlation between the resistances to the two soilborne pathogens Pu or Gt and the abundance of *Pseudomonas* harboring antimicrobial genes in the investigated agricultural soils (**Figure 7**). The lack of a significant positive correlation between disease resistance and abundance of antimicrobial pseudomonads could also be because only the *phlD*^+^ bacteria were detected at more than 10^5^ cells/g root, which is close to the threshold considered to be relevant for biocontrol activity (Raaijmakers et al., 1999; Haas and Défago, 2005) in many soils. By analogy, the populations of PHZ and PRN producing pseudomonads might therefore also be too low to contribute to efficient pathogen suppression in the soils of our study. Although correlations between gene abundances and disease resistance were never significant, it is still worth to note that for both pathogens they were mostly positive (**Figure 7A**). The expression of antimicrobial genes by reporter strains cannot be directly correlated to pathogen suppression data in the present study. Nevertheless, our findings suggest that high expression levels of antimicrobial genes in a particular soil may not necessarily be indicative of high levels of pathogen suppression (**Figure 6**). Furthermore, soils supporting the high expression of antimicrobial genes mostly harbored lower numbers of antimicrobials-producing pseudomonads (**Figure 6**). We thus speculate that the specific biotic and abiotic factors operating in the distinct agricultural soils might differently influence the abundance and expression of antimicrobial genes. At the present stage, it remains therefore elusive to which extent and how exactly pseudomonads producing DAPG, PHZ, and PRN contribute to the disease resistance of the investigated agricultural soils. Moreover, only two soilborne pathogens were investigated here and individual soils strongly differed in their response to Pu and Gt. It is likely that different relations to gene abundances and expression would be found for other pathogen and plant species.

Soil nutrients are known to be important factors influencing resistance to pathogens (Martin and Hancock, 1986; Löbmann et al., 2016) and the abundance of pseudomonads producing antimicrobial compounds (Meyer et al., 2010; Kim et al., 2013). Recently, Löbmann et al. (2016) found that soils can influence the resistance to Pu in two ways: through abiotic effects that inhibit pathogen growth, which was the case in soils with a high pH, high calcium, and high clay content; or through balanced nutrient contents, which were hypothesized to stimulate the proliferation of plant-beneficial microorganisms. Löbmann et al. (2016) postulated this effect in soils with high P, K, Mg, sand and organic matter contents, although the involved plant-beneficial microorganisms were not identified in their study. In our study, we found significant positive correlations between Pu resistance and the contents of nitrate and Mg in soil. However, the same two nutrients were also positively correlated with the abundance of the DAPG biosynthesis gene *phlD*, so we cannot conclude on how exactly these nutrients have a positive impact on soil resistance. They could have a negative effect on the pathogen, have a positive effect on the growth of the plants and increase their pathogen resistance, or have an indirect positive effect on soil resistance by promoting beneficial bacteria.

We found no significant correlation between pH and abundance of *Pseudomonas* harboring antimicrobial genes, probably because the soils of this study all had a pH close to neutral and ranged from 6.0 (Cadenazzo) to 7.7 (Cazis) (**Table 1**). The influence of pH of agricultural soils on the population sizes of pseudomonads producing antimicrobial compounds is poorly understood. Previously, indigenous pseudomonads were found to be more abundant in soils with a neutral pH than in soils with an acidic pH (Kim et al., 2013). Likewise, *P. protegens* CHA0 inoculated into soils with an acidic pH reached lower population sizes compared to when it was inoculated into soils with neutral or basic pH (Mascher et al., 2013).

We obtained contrasting results for clay and silt, where clay was positively and silt negatively correlated with gene abundance (**Figure 8**). For gene expression, the opposite was the case. The precise relation between *Pseudomonas* abundance and clay content is not known. However, high clay content in soil was previously shown to reduce the expression of the HCN biosynthetic gene *hcnC* (Novinscak and Filion, 2011) and the biocontrol activity of phenazine-producing strains (Ownley et al., 2003). The pronounced effect of clay on biocontrol activity of pseudomonads was also observed in studies carried out in artificial soils (Keel et al., 1989; Almario et al., 2013b, 2014). In particular, vermiculitic clay supported a higher level of biocontrol activity and HCN production than illitic clay (Keel et al., 1989), and the expression of *phlA* was greater in the presence of vermiculite than in the presence of illite (Almario et al., 2013b).

The plant macronutrients nitrate, potassium and magnesium also inversely influenced abundance and expression of antimicrobial genes. To the best of our knowledge, it has not been investigated if high macronutrient contents in the soil directly stimulate the growth of antimicrobial pseudomonads. We hypothesize that the positive correlation between abundance of antimicrobial pseudomonads and certain macronutrients such as nitrate could also be due to an indirect effect, e.g., via stimulation of plant growth and increased root exudation. Support for this hypothesis comes from a recent study on maize roots, where nitrogen concentration in soil was found to be positively correlated with plant root exudation and abundance of rhizosphere bacteria (Zhu et al., 2016). For the micronutrients, a significant, though negative, correlation was found only for manganese and the expression of the HCN and PHZ biosynthetic genes (**Figure 8**). However, in previous studies, several micronutrients were described as factors that affect abundance, gene expression, metabolite production and biocontrol activity in pseudomonads. For instance, copper negatively influenced the abundance of *Pseudomonas* spp. in agricultural soils (Brandt et al., 2006), while the bioavailability of iron affected the expression of *phlA* and the production of HCN in artificial soils (Keel et al., 1989; Almario et al., 2013b). The biocontrol activity of phenazine-producing strains was positively correlated to zinc and negatively to iron and manganese levels in soil (Ownley et al., 2003). Moreover, the production of several antimicrobial metabolites, notably DAPG, pyoluteorin and PRN by strain *P. protegens* CHA0 was stimulated by zinc, but these experiments were performed in culture media (Duffy and Défago, 1997, 1999). The lack of significant correlations between micronutrient contents in soil and abundance and expression of antimicrobial genes in our study could be due to the relatively high micronutrient content in the sampled soils. In fact, all studied soils had sufficient amounts of micronutrients according to the classification for farmers approved by the Swiss government (Flisch et al., 2009). Boron concentration was an exception, as three out of 10 sampled soils tested as poor (**Table 1**).

## Conclusion

Results of this study suggest that resistance of soils to pathogens, and abundance and expression of antimicrobial genes are not generally positively or negatively correlated in a wide range of diverse agricultural soils. Complex interactions depending on the host–pathogen system and the soil composition determine pathogen resistance of soils and the abundance and expression of antimicrobial genes. The abundance of antimicrobial metabolites producing pseudomonads in the investigated agricultural soils and the expression of biosynthetic genes for these compounds as studied here using reporter strains seem to be differentially shaped by multiple soil factors. This could explain, at least in part, why soils that sustain high numbers of these bacteria, often support only low levels of antimicrobial gene expression and vice versa. Therefore, to better understand the links between soil characteristics and abundance and expression of antimicrobial genes in pseudomonads, future studies should include extreme soils (i.e., highly acidic or alkaline soils or soils enriched in or depleted of specific nutrients). Pseudomonads are probably only one among many microbial groups determining the natural pathogen tolerance or resistance of agricultural soils (Mendes et al., 2011; Kyselkova et al., 2014; Cha et al., 2016; Raaijmakers and Mazzola, 2016). In future work, the potential of *Pseudomonas* bacteria as bio-indicators of soil resistance has to be re-evaluated with care. It will probably be difficult to identify specific groups of microorganisms as general indicators of soil health and disease resistance, since natural disease suppression likely requires individual compositions of the beneficial microbiota depending on the soil type, the crop species, the soilborne disease and maybe even the cropping system.

## Author Contributions

NI, FD, MM, and CK designed the research. MM and CK supervised the study. MF and FM organized the soil sampling and soil analysis. FD performed pathogen resistance greenhouse experiments. FD, DM, and OM developed the qPCR assays. FD performed the DNA extractions and qPCR assays. NI developed the reporter strains and performed the gene expression experiments. FD and NI analyzed the data. EB, JS, MW, and CV assisted with experiments and/or data evaluation. FD, NI, MM, and CK wrote the manuscript. All authors critically revised the manuscript and approved the final version.

## Conflict of Interest Statement

The authors declare that the research was conducted in the absence of any commercial or financial relationships that could be construed as a potential conflict of interest.

## Footnotes

1
http://www.meteoswiss.admin.ch/home/climate/past/homogenous-monthly-data.html

2
https://www.ncbi.nlm.nih.gov/genbank/
