# Supplementary material for: Relationships between Root Pathogen Resistance, Abundance and Expression of Pseudomonas Antimicrobial Genes, and Soil Properties in Representative Swiss Agricultural Soils
Source: Front Plant Sci. 2017 Mar 29;8:427. doi: 10.3389/fpls.2017.00427 (PMC5372754; doi:10.3389/fpls.2017.00427)
Supplement: Supplementary file 1 [file Data_Sheet_1.PDF]

Supplementary Tables and Figures

Relationships between root pathogen resistance, abundance and expression of *Pseudomonas* antimicrobial genes, and soil properties in representative Swiss agricultural soils

Nicola Imperiali, Francesca Dennert, Jana Schneider, Titouan Laessle, Christelle Velatta, Marie Fesselet, Michele Wyler, Fabio Mascher, Olga Mavrodi, Dmitri Mavrodi, Monika Maurhofer, and Christoph Keel

Correspondence:

Christoph Keel: christoph.keel@unil.ch

Monika Maurhofer: monika.maurhofer@usys.ethz.ch

Contents:

Table S1. Strains used for specificity testing of qPCR assays and for *in-vivo* standard curves.

Table S2. Efficiencies and detection limits of qPCR assays using *in vivo* standard curves for quantification

Table S3. Antimicrobial gene expression at the single cell level and root colonization by GFP-marked reporters of *Pseudomonas protegens* (CHA0-*gfp*) or *Pseudomonas chlororaphis* (PCL1391-*gfp*) carrying *mcherry*-based fusions to *phlA*, *hcnA*, *prnA* and *phzA*, respectively, in soils from 10 Swiss fields planted with wheat.

Figure S1. Relative resistance of 10 representative Swiss agricultural soils planted with spring wheat to increasing inoculum concentrations of *Gaeumannomyces tritici*.

Figure S2. Relative resistance of 10 representative Swiss agricultural soils planted with cucumber to increasing inoculum concentrations of *Pythium ultimum*.

Figure S3. Abundance of resident *Pythium ultimum* on roots of spring wheat grown in 10 Swiss agricultural soils.

Figure S4. Comparison between most probable number PCR (MPN-PCR) and qPCR for the determination of the abundance of *phlD*$^+$ and *phzF*$^+$ cells on roots of spring wheat.

TABLE S1 | Strains used for specificity testing of qPCR assays and for *in-vivo* standard curves.

Strain	Antimicrobial metabolites produced by strain[1]	Amplification with PhzF_2Fm/ PhzF_2Rm	Amplification with PhlD_65F_DEG/ PhlD_236R_DEG	*In vivo* standard curve[2]	Origin of strain	Reference
**Diverse soil bacteria**						
Brevibacterium iodinum ATCC 15728	PHZ	-[3]	nd[3]		AmericanType Culture Collection[4]	Turner and Messenger (1986)
Burkholderia cepacia ATCC 25416	PHZ, PRN	-	nd	*prnD*	Onion, USA	Mahenthiralingam et al. (2000)
Burkholderia lata ATCC 17760	PHZ	-	nd		American Type Culture Collection	American Type Culture Collection
Burkholderia sp. 5.5B5	PHZ	-	nd		Soil, USA	Cartwright et al. (1995)
Burkholderia thailandensis DSM 13276	PHZ, PRN	nd[3]	nd	*prnD*	Environmental sample, Thailand	Brett et al. (1998)
Pectobacterium carotovorum subsp. *carotovorum* CRI 1043 (BAA-672)	PHZ	-	nd		Potato, Scotland	Bull et al. (1994)
Pectobacterium carotovorum subsp. *carotovorum* cc303	PHZ	-	nd		Soil, Oregon	Bull et al. (1994)
***Pseudomonas aeruginosa* lineage[5]**						
Pseudomonas aeruginosa ATCC 23993	PHZ	-	nd		Clinical sample, Japan	American Type Culture Collection
Pseudomonas aeruginosa ATCC 25011	PHZ	-	nd		Clinical sample, Japan	American Type Culture Collection
Pseudomonas aeruginosa PAO1	PHZ	-	-[3]		Clinical sample, Australia	Pseudomonas Genetic Stock Centre[6]
***Pseudomonas fluorescens* lineage[5]**						
Pseudomonas aridus R1-43-08 rif	PHZ	+[3]	nd		Wheat rhizosphere, WA, USA	Parejko et al. (2013)
Pseudomonas aureofaciens 30-84	PHZ	+	-	*phzF*	Wheat rhizosphere, USA	Thomashow et al. (1990)
Pseudomonas brassicacearum TM1A3	DAPG	nd	+[3]	*phlD*	Tomato rhizosphere, Switzerland	Keel et al. (1996)
Pseudomonas cerealis L1-45-08 rif	PHZ	+	-	*phzF*	Wheat rhizosphere, WA, USA	Parejko et al. (2013)
Pseudomonas cerealis R5-89-07	PHZ	+	nd		Wheat rhizosphere, WA, USA	Mavrodi et al. (2010)
Pseudomonas chlororaphis LMG 5004	PHZ, PRN	nd	nd	*prnD*	Plate contaminant	Peix et al. (2007)
Pseudomonas chlororaphis 1391	PHZ	+	-		Tomato rhizosphere, Spain	Chin-A Woeng et al. (1998)
Pseudomonas chlororaphis ATCC 17411	PHZ	+	nd		American Type Culture Collection	Turner and Messenger (1986)

Pseudomonas chlororaphis ATCC 17809	PHZ	+	nd		American Type Culture Collection	Stanier et al. (1966)
Pseudomonas chlororaphis ATCC 9446	PHZ	+	nd		Plate contaminant	American Type Culture Collection
Pseudomonas chlororaphis BS1393	PHZ	+	nd		Soil, Russia	All-Russian Collection of Microorganisms[7]
Pseudomonas chlororaphis CD	PHZ	+	-		Cyclops	Ruffner et al., (2015)
Pseudomonas chlororaphis PGS12	PHZ	+	nd		Corn rhizosphere, USA	Georgakopoulos (1994)
Pseudomonas chlororaphis TX-1	PHZ	+	nd		Turfgrass soil, Michigan	Eco Soils Systems, San Diego, USA
Pseudomonas corrugata DSM 7228T	none	nd	-		Tomato stem, UK	Scarlett et al. (1978)
Pseudomonas brassicacearum F113	DAPG	nd	+	*phlD*	Sugarbeet rhizosphere, Ireland	Shanahan et al. (1992)
Pseudomonas fluorescens Q12-87	DAPG	nd	+		Wheat rhizosphere, WA, USA	Keel et al. (1996)
Pseudomonas fluorescens Q128-87	DAPG	nd	+		Wheat rhizosphere, WA, USA	Harrison et al. (1993)
Pseudomonas synxantha 2-79	PHZ	+	-	*phzF*	Wheat rhizosphere, WA, USA	Weller and Cook (1983)
Pseudomonas fluorescens C*1A1	DAPG	nd	+		Cucumber rhizosphere, Switzerland	Keel et al. (1996)
Pseudomonas fluorescens P97.38	DAPG	nd	+		Cucumber rhizosphere, Switzerland	Wang et al. (2001)
Pseudomonas fluorescens Q37-87	DAPG	nd	+		Wheat rhizosphere, WA, USA	Keel et al. (1996)
Pseudomonas fluorescens Q65c-80	DAPG	nd	+		Wheat rhizosphere, WA, USA	Harrison et al. (1993)
Pseudomonas fluorescens Q86-87	DAPG	nd	+		Wheat rhizosphere, WA, USA	Keel et al. (1996)
Pseudomonas kilonensis DSM 13647	DAPG	nd	+		Agricultural soil, Germany	Sikorsky et al. (2001)
Pseudomonas kilonensis P12	DAPG	nd	+		Tobacco rhizosphere, Switzerland	Keel et al. (1996)
Pseudomonas orientalis L1-3-08 rif	PHZ	+	nd		Wheat rhizosphere, WA, USA	Parejko et al. (2013)
Pseudomonas protegens PF	DAPG, PRN	nd	+		Wheat leaves, USA	Levy et al. (1992)
Pseudomonas protegens BRIP	DAPG; PRN	nd	+		Cyclops, Switzerland	Ruffner et al. (2015)
Pseudomonas protegens CHA0	DAPG, PRN	-	+	*phlD*, *prnD*	Tobacco black root rot suppressive soil, Morens, Switzerland	Stutz et al. (1986)
Pseudomonas protegens Pf-5	DAPG, PRN	-	+		Texas, USA	Howell and Stipanovic (1980)
Pseudomonas protegens PGNR1	DAPG, PRN	nd	+		Tobacco rhizosphere, Ghana	Keel et al. (1996)
Pseudomonas sp. R11-45-07	PHZ	+	nd		Wheat rhizosphere, WA, USA	Mavrodi et al. (2010)
Pseudomonas sp. R2-7-07	PHZ	+	nd		Wheat rhizosphere, WA, USA	Mavrodi et al. (2010)
Pseudomonas sp R4-34-07	PHZ	+	nd		Wheat rhizosphere, WA, USA	Mavrodi et al. (2010)
Pseudomonas sp. R4-35-07	PHZ	+	nd		Wheat rhizosphere, WA, USA	Mavrodi et al. (2010)
Pseudomonas sp. C1Phz19	PHZ	+	nd		Fusarium wilt suppressive soil, Châteaurenard, France	Mavrodi et al. (2010)
Pseudomonas sp. CMR12a	PHZ, DAPG	+	+		Cocoyam rhizosphere, Cameroon	Perneel et al. (2007)
Pseudomonas sp. CMR5c	PHZ, DAPG	+	+	*prnD*	Cocoyam rhizosphere, Cameroon	Perneel et al. (2007)
Pseudomonas sp. L1-11-07	PHZ	+	nd		Wheat rhizosphere, WA, USA	Parejko et al. (2013)
Pseudomonas sp. PILH1	DAPG	nd	+		Tomato rhizosphere, Italy	Keel et al. (1996)

Pseudomonas sp. R11-23-07	PHZ	+	nd	Wheat rhizosphere, WA, USA	Parejko et al. (2013)
Pseudomonas sp. R5-89-07	PHZ	+	nd	Wheat rhizosphere, WA, USA	Mavrodi et al. (2010)
Pseudomonas sp. R5-90-07	PHZ	+	nd	Wheat rhizosphere, WA, USA	Mavrodi et al. (2010)
Pseudomonas sp. SLPH 10	PHZ	+	nd	Take all decline soil, the Netherlands	Jos M. Raaijmakers[8]
Pseudomonas sp. R14-24-07	PHZ	+	nd	Wheat rhizosphere, WA, USA	Mavrodi et al. (2010)
Pseudomonas thivervalensis DSM 13194	DAPG	nd	+	Rapeseed, France	Achouak et al. (2000)
Pseudomonas thivervalensis PITR2	DAPG	nd	+	Wheat rhizosphere, Italy	Keel et al. (1996)

[1]Only the metabolites of interest for this study, i.e. phenazines (PHZ), 2,4-diacetylphloroglucinol (DAPG), and pyrrolnitrin (PRN) are indicated. Many of these strains produce additional antimicrobial metabolites. For more detailed information, see the reference of each strain and Flury et al. (2016).
[2]Indicated strains were selected to generate in-vivo standard curves for phlD, phzF and prnD qPCR used for the quantification of DAPG, PHZ and PRN biosynthesis genes.
[3]Legend: += amplification, -= no amplification, nd= not determined.
[4]American Type Culture Collection, 10801 University Blvd, Manassas, VA 20110, USA.
[5]Phylogeny according to Mulet et al., (2010).
[6]Pseudomonas Genetic Stock Centre, East Carolina University, Greenville, NC 27858-4353, USA.
[7]All-Russian Collection of Microorganisms, Russia, 142290, Moscow Region, Pushchino, pr. Nauki, 5, IBPM.
[8]Kindly provided by Jos M. Raaijmakers, Netherlands Institute of Ecology, Wageningen, The Netherlands.

Achouak, W., Sutra, L., Heulin, T., Meyer, J.-M., Fromin, N., Degraeve, S., et al. (2000). *Pseudomonas brassicacearum* sp. nov. and *Pseudomonas thivervalensis* sp. nov., two root-associated bacteria isolated from *Brassica napus* and *Arabidopsis thaliana*. *Int. J. Syst. Evol. Microbiol.* 50, 9-18.

Brett, P.J., DeShazer, D., and Woods, D.E. (1998). *Burkholderia thailandensis* sp. nov., a *Burkholderia pseudomallei*-like species. *Int. J. Syst. Bacteriol.* 48, 317-320.

Bull, C.T., Ishimaru, C.A., and Loper, J.E. (1994). Two genomic regions involved in catechol siderophore production by *Erwinia carotovora*. *Appl. Environ. Microbiol.* 60, 662-669.

Cartwright, D.K., Chilton, W.S., and Benson, D.M. (1995). Pyrrolnitrin and phenazine production by *Pseudomonas cepacia*, strain 5.5B, a biocontrol agent of *Rhizoctonia solani*. *Appl. Microbiol. Biotechnol.* 43, 211-216.

Chin-A-Woeng, T.F., Bloemberg, G.V., van der Bij, A.J., van der Drift, K.M., Schripsema, J., Kroon, B., et al. (1998). Biocontrol by phenazine-1-carboxamide-producing *Pseudomonas chlororaphis* PCL1391 of tomato root rot caused by *Fusarium oxysporum* f. sp. *radicis-lycopersici*. *Mol. Plant-Microbe Interact.* 11, 1069-1077.

Georgakopoulos, D.G., Hendson, M., Panopoulos, N.J., and Schroth, M.N. (1994). Cloning of a phenazine biosynthetic locus of *Pseudomonas aureofaciens* PGS12 and analysis of its expression in vitro with the ice nucleation reporter gene. *Appl. Environ. Microbiol.* 60, 2931-2938.

Flury, P., Aellen, N., Ruffner, B., Pechy-Tarr, M., Fataar, S., Metla, Z., et al. (2016). Insect pathogenicity in plant-beneficial pseudomonads: phylogenetic distribution and comparative genomics. *ISME J* 10.1038/ismej.2016.5**,** 1-16. doi: 10.1038/ismej.2016.5.

Harrison, L.A., Letendre, L., Kovacevich, P., Pierson, E., and Weller, D. (1993). Purification of an antibiotic effective against *Gaeumannomyces graminis* var. *tritici* produced by a biocontrol agent, *Pseudomonas aureofaciens*. *Soil Biol. Biochem.* 25, 215-221.

Howell, C., and Stipanovic, R. (1980). Suppression of *Pythium ultimum* induced damping off of cotton seedlings by *Pseudomonas fluorescens* and its antibiotic pyoluteorin. *Phytopathology* 70, 712-715.

Keel, C., Weller, D., Natsch, A., Défago, G., Cook, J.R., and Thomashow, L. (1996). Conservation of the 2,4-diacetylphloroglucinol biosynthesis locus among fluorescent *Pseudomonas* strains from diverse geographic locations. *Appl. Environ. Microbiol.* 62, 552-563.

Levy, E., Gough, F., Berlin, K., Guiana, P., and Smith, J. (1992). Inhibition of *Septoria tritici* and other phytopathogenic fungi and bacteria by *Pseudomonas fluorescens* and its antibiotics. *Plant Pathol.* 41, 335-341.

Mahenthiralingam, E., Coenye, T., Chung, J.W., Speert, D.P., Govan, J.R.W., Taylor, P., et al. (2000). Diagnostically and experimentally useful panel of strains from the *Burkholderia cepacia* complex. *J. Clin. Microbiol.* 38, 910-913.

Mavrodi, D.V., Peever, T.L., Mavrodi, O.V., Parejko, J.A., Raaijmakers, J.M., Lemanceau, P., et al. (2010). Diversity and evolution of the phenazine biosynthesis pathway. *Appl. Environ Microbiol.* 76, 866-879.

Mulet, M., Lalucat, J., and Garcia-Valdes, E. (2010). DNA sequence-based analysis of the Pseudomonas species. *Environ Microbiol* 12(6)**,** 1513-1530. doi: 10.1111/j.1462-2920.2010.02181.x.

Parejko, J.A., Mavrodi, D.V., Mavrodi, O.V., Weller, D.M., and Thomashow, L.S. (2013). Taxonomy and distribution of phenazine-producing *Pseudomonas* spp. in the dryland agroecosystem of the inland Pacific Northwest, United States. *Appl. Environ. Microbiol.* 79, 3887-3891.

Peix, A., Valverde, A., Rivas, R., Igual, J.M., Ramírez-Bahena, M.-H., Mateos, P.F., et al. (2007). Reclassification of *Pseudomonas aurantiaca* as a synonym of *Pseudomonas chlororaphis* and proposal of three subspecies, *P. chlororaphis subsp*. *chlororaphis* subsp. nov., *P. chlororaphis* subsp. *aureofaciens* subsp. nov., comb. nov. and *P. chlororaphis* subsp. *aurantiaca* subsp. nov., comb. nov. *Int. J. Syst. Evol. Microbiol.* 57, 1286-1290.

Perneel, M., Heyrman, J., Adiobo, A., De Maeyer, K., Raaijmakers, J.M., De Vos, P., et al. (2007). Characterization of CMR5c and CMR12a, novel fluorescent *Pseudomonas* strains from the cocoyam rhizosphere with biocontrol activity. *J. Appl. Microbiol.* 103, 1007-1020.

Ruffner, B., Pechy-Tarr, M., Höfte, M., Bloemberg, G., Grunder, J., Keel, C., et al. (2015). Evolutionary patchwork of an insecticidal toxin shared between plant-associated pseudomonads and the insect pathogens *Photorhabdus* and *Xenorhabdus*. *BMC Genomics* 16, 609.

Scarlett, C.M., Fletcher, J., Roberts, P., and Lelliott, R. (1978). Tomato pith necrosis caused by *Pseudomonas corrugata* n. sp. *Ann. Appl. Biol.* 88, 105-114.

Shanahan, P., O'Sullivan, D.J., Simpson, P., Glennon, J.D., and O'Gara, F. (1992). Isolation of 2,4-diacetylphloroglucinol from a fluorescent pseudomonad and investigation of physiological parameters influencing its production. A*ppl. Environ. Microbiol.* 58, 353-358.

Sikorski, J., Stackebrandt, E., and Wackernagel, W. (2001). *Pseudomonas kilonensis* sp. nov., a bacterium isolated from agricultural soil. *Int. J. Syst. Evol. Microbiol.* 51, 1549-1555.

Stanier, R.Y., Palleroni, N.J., and Doudoroff, M. (1966). The aerobic pseudomonads a taxonomic study. *Microbiology* 43, 159-271.

Stutz, E.W., Défago, G., and Kern, H. (1986). Naturally occurring fluorescent pseudomonads involved in suppression of black root rot of tobacco. *Phytopathology* 76, 181-185.

Thomashow, L.S., Weller, D.M., Bonsall, R.F., and Pierson, L.S. (1990). Production of the antibiotic phenazine-1-carboxylic acid by fluorescent *Pseudomonas* species in the rhizosphere of wheat. *Appl. Environ. Microbiol.* 56, 908-912.

Turner, J.M., and Messenger, A.J. (1986). "Occurrence, biochemistry and physiology of phenazine pigment production," in *Advances in Microbial Physiology*, eds. A.H. Rose and D.W. Tempest (Academic Press), 211-275.

Wang, C., Ramette, A., Punjasamarnwong, P., Zala, M., Natsch, A., Moënne-Loccoz, Y., et al. (2001). Cosmopolitan distribution of *phlD*-containing dicotyledonous crop-associated biocontrol pseudomonads of worldwide origin. *FEMS Microbiol. Ecol.* 37, 105-116.

Weller, D., and Cook, R. (1983). Suppression of take-all of wheat by seed treatments with fluorescent pseudomonads. *Phytopathology* 73, 463-469.

TABLE S2 | Efficiencies and detection limits of qPCR assays using *in vivo* standard curves for quantification

Target[1]	Efficiency (%)[2]	Slope	R^2	Detection limit (gene copies/g root dry weight)[3]
phlD	92.19	-3.525	0.992	2,000
phzF	110.56	-3.090	0.987	200
prnD	86.07	-3.708	0.900	2,000

[1]*Mixtures of strains containing the target genes were used. The strains used for each in vivo standard curve are indicated in Table S1. The target concentrations ranged from* 10^8 *cells per gram of root to 0 cells per gram of root (noninoculated control) in tenfold dilutions (see Materials and Methods). The quantity of template used per reaction was 2 µl of undiluted DNA extracted from rhizosphere wash.*
[2]*Efficiency*$=10^{(-1/slope)}-1$
[3]*Three biological replicates were used for each concentration.*

TABLE S3 | Antimicrobial gene expression at the single cell level and root colonization by GFP-marked reporters of *Pseudomonas protegens* (CHA0-*gfp*) or *Pseudomonas chlororaphis* (PCL1391-*gfp*) carrying *mcherry*-based fusions to *phlA*, *hcnA*, *prnA* and *phzA*, respectively, in soils from 10 Swiss fields planted with wheat[1].

Soil[1]	*P. protegens* CHA0-*gfp*				*P. chlororaphis* PCL1391-gfp	
	***phlA* expression (RFU per cell) (× 10^2)**	***hcnA* expression (RFU per cell) (× 10^2)**	***prnA* expression (RFU per cell) (× 10^2)**	**Number of cells g^{-1} dry root (× 10^7)**	***phzA* expression (RFU per cell) (× 10^2)**	**Number of cells g^{-1} dry root (× 10^7)**
Cd	49.9 a	3.18 a	1.77 a	1.88 ab	7.04 a	2.42 a
Cx	38.4 ab	1.72 cde	0.86 c	0.40 def	4.91 ab	0.55 de
Cz	28.4 cde	1.26 def	0.95 c	1.93 ab	4.50 abc	0.76 cd
De	36.2 abc	4.82 a	1.65 ab	1.39 cde	2.59 d	0.90 c
Es	32.6 bcd	3.00 ab	1.73 ab	0.29 g	4.04 abc	0.31 e
Gr	15.9 fg	1.75 cde	1.48 b	0.35 fg	3.23 c	0.96 bc
Ta	29.1 de	1.96 bc	1.65 ab	0.58 efg	2.59 d	0.34 de
Ut	12.7 g	1.26 g	1.86 a	0.85 bcd	2.66 d	1.79 ab
Vo	25.3 ef	1.75 ef	1.06 c	2.41 a	3.51 bc	1.92 a
Wi	15.6 g	1.32 g	0.99 c	1.09 bc	3.44 bc	2.17 a

[1]*Expression and root colonization by reporter strains was monitored by fluorescence-activated cell-sorting-based flow cytometry using CHA0-gfp carrying plasmids pME9012 (phlA-mcherry), pME9011 (hcnA-mcherry), or pME11011 (prnA-mcherry) or PCL1391-gfp carrying pME11017 (phzA-mcherry). Seedlings inoculated with the reporter strains were grown in soil microcosms for five days prior to analysis of bacterial cells in root washes. Data on expression are shown as relative fluorescence units (RFU) and represent the median mCherry expression per GFP-tagged Pseudomonas cell. Root colonization levels were recorded as number of GFP-tagged cells per gram of dry root. Results from three independent experiments with nine replicates each are presented. Letters indicate significant differences (Dunn test, $p<0.05$).*

[2]*Soils were sampled at the following field sites: Cd, Cadenazzo; Cx, Courtedoux; Cz, Cazis; De, Delley; Es, Eschikon; Gr, Grangeneuve; Ta, Taenikon; Ut, Utzenstorf; Vo, Vouvry; Wi, Witzwil.*

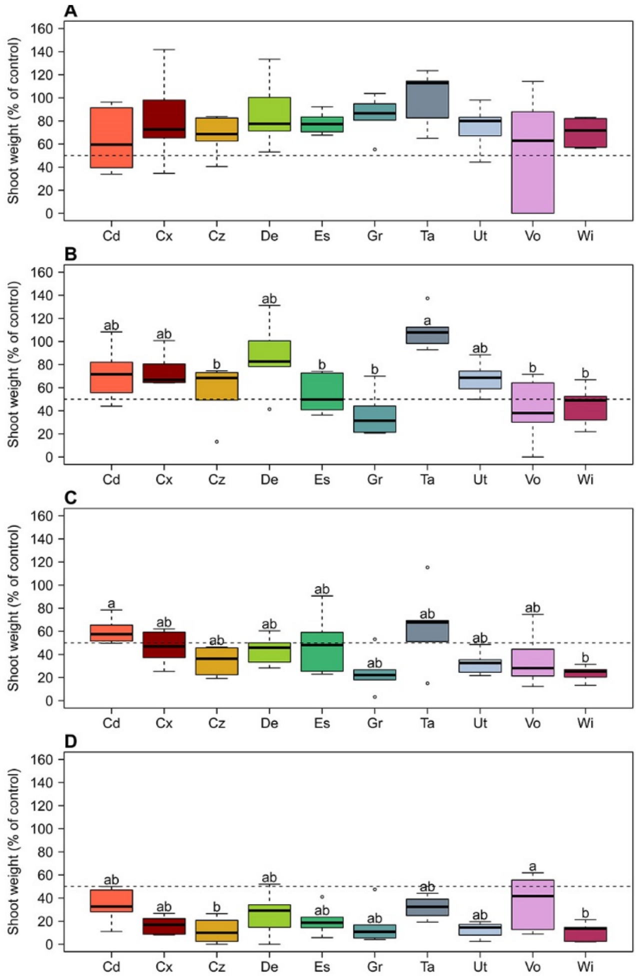

FIGURE S1 | Relative resistance of 10 representative Swiss agricultural soils planted with spring wheat to increasing inoculum concentrations of *Gaeumannomyces tritici*. Inoculum concentrations per pot were **(A)** 0.2 g, **(B)** 0.6 g, **(C)** 2.0 g, and **(D)** 6.0 g. Each pathogen concentration and soil was tested in six replicate pots. Soil resistance is shown as fresh shoot weight of plants in artificially pathogen-infested soil compared to shoot weight of control plants grown in non-infested soil. The dotted line indicates 50% of shoot weight compared to the control. Letters indicate significant differences (Kruskal-Wallis test, $p<0.05$). Sampling sites: Cd, Cadenazzo; Cx, Courtedoux; Cz, Cazis; De, Delley; Es, Eschikon; Gr, Grangeneuve; Ta, Taenikon; Ut, Utzenstorf; Vo, Vouvry; Wi, Witzwil.

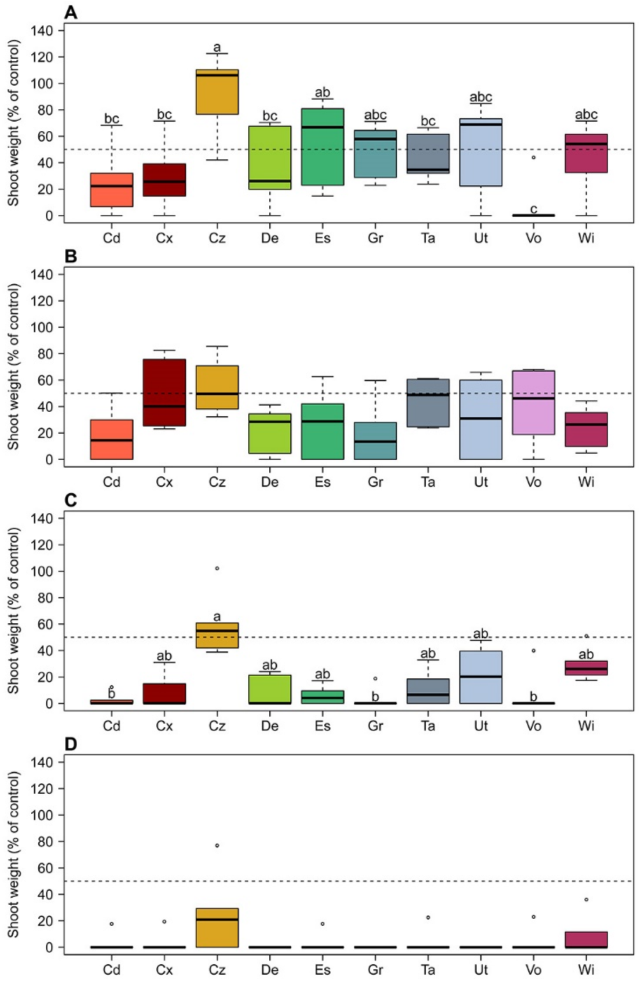

FIGURE S2 | Relative resistance of 10 representative Swiss agricultural soils planted with cucumber to increasing inoculum concentrations of *Pythium ultimum*. Inoculum concentrations per pot were **(A)** 0.125 g, **(B)** 0.25 g, **(C)** 0.5 g, and **(D)** 1.0 g. Each pathogen concentration and soil was tested in six replicate pots. Soil resistance is shown as fresh shoot weight of plants in artificially pathogen-infested soil compared to shoot weight of control plants grown in non-infested soil. The dotted line indicates 50% of shoot weight compared to the control. Letters indicate significant differences (Kruskal-Wallis test, $p<0.05$). Sampling sites: Cd, Cadenazzo; Cx, Courtedoux; Cz, Cazis; De, Delley; Es, Eschikon; Gr, Grangeneuve; Ta, Taenikon; Ut, Utzenstorf; Vo, Vouvry; Wi, Witzwil.

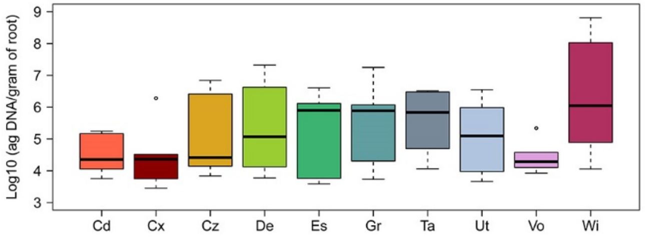

FIGURE S3 | Abundance of resident *Pythium ultimum* on roots of spring wheat grown in 10 Swiss agricultural soils. Abundance was determined with a qPCR assay targeting the internal transcribed spacer (ITS) rRNA gene region. For each soil, six replicates were used. The quantity of *P. ultimum* DNA per gram of roots is shown (ag, attogram). No significant differences between samples from different soils were found (Kruskal-Wallis test, p<0.05). Sampling sites: Cd, Cadenazzo; Cx, Courtedoux; Cz, Cazis; De, Delley; Es, Eschikon; Gr, Grangeneuve; Ta, Taenikon; Ut, Utzenstorf; Vo, Vouvry; Wi, Witzwil.

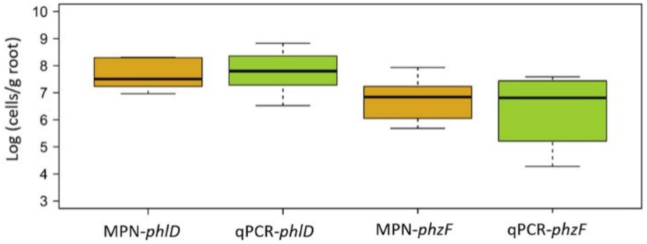

FIGURE S4 | Comparison between most probable number PCR (MPN-PCR) and qPCR for the determination of the abundance of *phlD*$^{+}$ and *phzF*$^{+}$ cells on roots of spring wheat. Six replicates of roots of wheat grown in Taenikon soil were used. The same primers (see Table 2) were used for MPN-PCR and qPCR. No significant difference between the two methods was detected.
